# Committed Human CD23-Negative Light-Zone Germinal Center B Cells Delineate Transcriptional Program Supporting Plasma Cell Differentiation

**DOI:** 10.3389/fimmu.2021.744573

**Published:** 2021-12-02

**Authors:** Kathleen Santamaria, Fabienne Desmots, Simon Leonard, Gersende Caron, Marion Haas, Céline Delaloy, Fabrice Chatonnet, Delphine Rossille, Amandine Pignarre, Céline Monvoisin, Marine Seffals, Claire Lamaison, Michel Cogné, Karin Tarte, Thierry Fest

**Affiliations:** ^1^ UMR 1236, University of Rennes 1, INSERM, Établissement Français du Sang Bretagne, Rennes, France; ^2^ Pôle de Biologie, Rennes University Medical Center, Rennes, France; ^3^ LabEx IGO “Immunotherapy, Graft, Oncology”, Nantes, France; ^4^ University of Rennes 1, UMS Biosit, H2P2 Platform, Rennes, France

**Keywords:** germinal center (GC), germinal center (GC) B cells, CD23+ B cells, B cell differentiation, plasmablasts/plasma cells, GC Light-Zone B cells

## Abstract

B cell affinity maturation occurs in the germinal center (GC). Light-zone (LZ) GC B cells (B_GC_-cells) interact with follicular dendritic cells (FDCs) and compete for the limited, sequential help from T follicular helper cells needed to escape from apoptosis and complete their differentiation. The highest-affinity LZ B_GC_-cells enter the cell cycle and differentiate into PCs, following a dramatic epigenetic reorganization that induces transcriptome changes in general and the expression of the *PRDM1* gene in particular. Human PC precursors are characterized by the loss of IL-4/STAT6 signaling and the absence of CD23 expression. Here, we studied the fate of human LZ B_GC_-cells as a function of their CD23 expression. We first showed that CD23 expression was restricted to the GC LZ, where it was primarily expressed by FDCs; less than 10% of tonsil LZ B_GC_-cells were positive. Sorted LZ B_GC_-cells left in culture and stimulated upregulated CD23 expression but were unable to differentiate into PCs – in contrast to cells that did not upregulate CD23 expression. An in-depth analysis (including single-cell gene expression) showed that stimulated CD23-negative LZ B_GC_-cells differentiated into plasmablasts and time course of gene expression changes delineates the transcriptional program that sustains PC differentiation. In particular, we identified a B cell proliferation signature supported by a transient *MYC* gene expression. Overall, the CD23 marker might be of value in answering questions about the differentiation of normal B_GC_-cells and allowed us to propose an instructive LZ B_GC_-cells maturation and fate model.

## Highlights

- Only human light-zone GC B cells that fail to express CD23 after appropriate stimulation are likely to differentiate into plasma cells
- Light-zone GC B cells heterogeneity through use of the CD23 marker allow to decipher gene expression changes during B cell differentiation

## Introduction

Within the secondary lymphoid organs, the germinal center (GC) is the primary site for the maturation of B-cell affinity. Iterative rounds of proliferation (associated with activation-induced cytidine (AID) enzyme activity) and positive selection of B-cell receptors (BCRs) with high affinity for their cognate antigens (Ags) ultimately lead to the production of memory B cells (MBCs) and plasma cells (PCs). Fully developed GCs comprise two functional zones, each of which contains a distinct GC B cell (B_GC_-cell) subtype. Firstly, the dark zone (DZ) is close to the T-zone and is where centroblasts proliferate in bursts. Secondly, the light zone (LZ) mainly contains non-proliferating centrocytes, some of them testing their BCR against the Ags displayed by follicular dendritic cells (FDCs) and thus competing for limited, sequential help from T follicular helper (Tfh) cells (1–3). Once the Ag is captured by the BCR, the cell receives a survival signal; the Ag is subsequently internalized, processed and presented on the cell surface as a class II MHC-peptide complex, which in turn leads to interaction with cognate Tfh cells. Hence, Tfh-derived signals enable B-cell proliferation, differentiation and isotype switching (3). MBCs tend to emerge earlier from a low-affinity compartment in the LZ, while PCs appear later during the immune response, committed B cells require strong Tfh cell help, and accumulate somatic hypermutation (4–6). Positively selected LZ B_GC_-cells escape from apoptosis, upregulate transiently their *MYC* expression, re-enter the cell cycle and travel to the DZ for further cell division and AID activity (7–9). It has been estimated that between 10% and 30% of the B cells that reach the LZ are selected and possibly re-enter the DZ; the remainder die mainly by apoptosis (9). Cognate B cell-T cell contact and help signal strength (both of which depend on BCR affinity) are likely to determine B cell fate. Medium-affinity cells express high levels of the BACH2 transcription factor and replenish the MBC pool, while the highest-affinity LZ B_GC_-cells are preferentially selected for cell cycle entry and differentiation into PCs (10, 11). With regard to this process, the results of a computational model of B_GC_-cell fate suggested that B cells that have been positively selected by successful Ag processing return to the DZ for asymmetric division, and that Ag affinity is inherited by only one of the daughter cells (12). On the other hand, PC precursors can exit the GC reaction *via* the DZ due to acquisition of CXCR4 expression leading cells to move to the CXCL12 rich DZ stromal environment. There is some experimental evidence to support this theory – notably the presence of PC precursors in the DZ (5, 13).

Committed B cells differentiate into plasmablasts (PBs) during an S phase in which specific oxidation of 5-methylcytosine (5mC) residues at given genomic positions leads to the expression of PC identity genes (14). Recently, we used an *in vitro* naïve B cell (NBC) differentiation model to demonstrate that human PC precursors are characterized by the loss of IL-4/STAT6 signaling and the absence of expression of CD23 [a pSTAT6-induced, low-affinity receptor for IgE (15)], although they are still imprinted by the previous IL-4 activation. In humans, low expression of the CD23 marker is reported in B_GC_-cells (16) and LZ B_GC_-cells (17), and the downregulation of CD23 is associated with PC commitment (18, 19). In mice, follicular B cells express CD23 (encoded by the *Fcer2a* gene), and CD23 expression is more intense on LZ follicular B_GC_-cells than on DZ B_GC_-cells (4, 20). In addition, B cell differentiation into PCs is accompanied by the loss of CD23 expression (21) and in contrast, recently reportedly that early positively selected cMyc^+^ murine LZ B_GC_-cells express the *Fcer2a* gene besides genes associated with both BCR signaling and immunological synapse suggesting a very recent activation following BCR engagement and GC B: Tfh interaction (20).

The objective of the present study was to assess the fate of human LZ B_GC_-cells as a function of their CD23 expression. In immune-histochemistry assessments, we determined the CD23 expression pattern in tonsillar GCs. Staining was restricted to the LZ, with strong expression by FDCs. By flow cytometry, we found less than 10% of tonsil LZ B_GC_-cells positive for CD23. However, most LZ B_GC_-cells expressed CD23 *in vitro* after productive Tfh cell help or appropriate cytokine stimulation. An in-depth analysis (including single-cell gene expression) showed that LZ B_GC_-cells CD23^+^ obtained after stimulation are unable to differentiate into PCs - unlike the cells that remain CD23^-^. Time course of single-cell gene expression changes during the differentiation of CD23^-^ LZ B_GC_-cells sheds new light on the final transcriptional switch that take place when human B cells metamorphosis into PBs characterized by the upregulation of *PRDM1* expression.

## Result

### In GCs, Only a Small Proportion of B Cells Expresses the CD23 Marker

**Figure 1:**
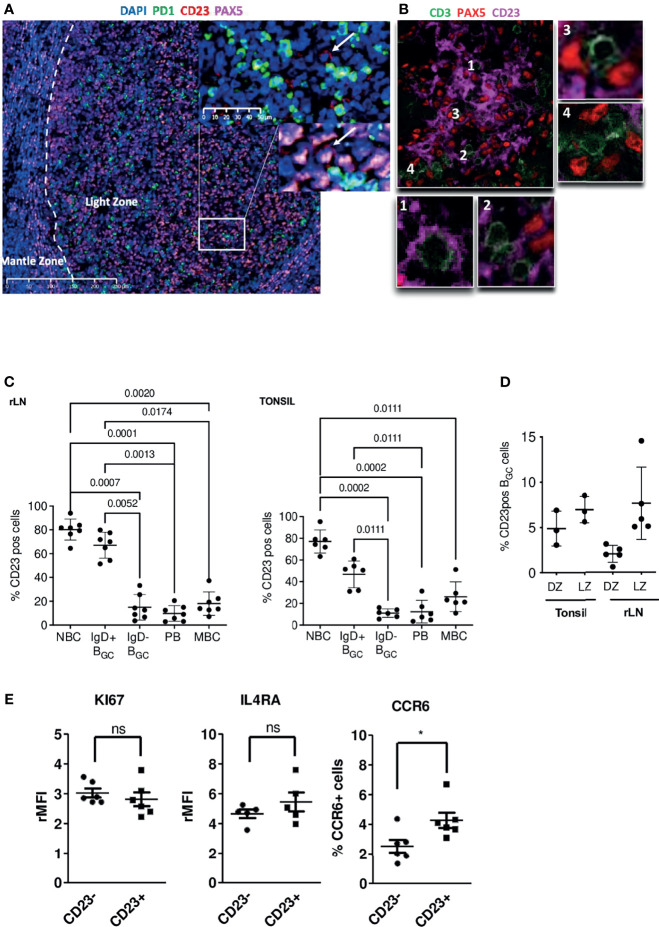
In the GC, CD23 is expressed in the LZ, mainly by FDCs but also by some B cells. **(A)** Immunofluorescence staining of CD23 (red), PAX5 (pink) and PD1 (green) in paraffin-embedded tonsil tissues; the white arrow shows a CD23^+^ B cell located in front of a PD1^+^ Tfh cell. **(B)** Immunofluorescence staining of CD23 (pink), PAX5 (red) and CD3 (green) on frozen tonsil tissues; the PAX5 staining pattern for B cells does not really match the CD23 marker; panels 1 & 2 show T cells surrounded by CD23^+^ dendritic extensions, and panels 3 & 4 show T cells in the vicinity of CD23^-^ B cells. **(C)** The proportion of CD23-expressing B cells was determined by flow cytometry in different B cell populations in tonsils (Right panel) and rLNs (Left panel): CD19^+^CD38^-^IgD^+^ naïve B cells (NBCs), CD19^+^CD38^+^IgD^+^ & IgD^-^ B_GC_-cells, CD19^+^CD38^++^IgD^-^ plasmablasts (PBs), and CD19^+^CD38^-^IgD^-^CD27^+^ memory B cells (MBCs) (one-way Anova & Kruskal-Wallis multiple comparisons test). **(D)** The proportion of CD23-positive cells in CXCR4^hi^CD83^lo^ DZ B_GC_-cells and CXCR4^lo^CD83^hi^ LZ B_GC_-cells in tonsils and rLNs. **(E)** Flow cytometry analysis of KI67, IL4RA and CCR6 in tonsil CD23^+^ and CD23^-^ LZ B_GC_-cells. Results are expressed as the relative mean fluorescence intensity (rMFI) or the percentage of positive cells (*P ≤ .05; "ns" for non significant; Mann-Whitney test).

To determine the CD23 cell surface marker’s expression patterns, sections of human tonsil tissue were stained with several combinations of antibodies in immunohistofluorescence experiments. In GCs, CD23 staining was restricted to the LZ. In line with the literature data (22, 23), CD21L^+^ FDCs were intensely stained (**Supplemental Figure S1A**). Using antibodies against CD23, PAX5 and PD1, we possibly distinguished CD23^+^ LZ B_GC_-cells located near PD1^+^ Tfh but without certainty due to the FDC labeling (**Figure 1A** and **Supplemental Figure S1B**). Indeed, CD23 staining of frozen tissue sections showed that the FDCs’ extensions formed a dense mesh around B and T cells (**Figure 1B** and **Supplemental Figure S1C**). Overall, our data show that FDCs in LZ are strongly positive for CD23, which makes it harder to detect CD23^+^ B_GC_-cells, in contrast to NBCs in the mantle zone. (**Supplemental Figure S1A**).

To determine the proportion of CD23^+^ B cells in GCs, we used flow cytometry to analyze cell suspensions obtained from tonsils and reactive lymph nodes (rLNs). Unlike NBCs and IgD^+^ B_GC_-cells, IgD^-^ B_GC_-cells, PBs and MBCs were predominantly CD23-negative (**Figure 1C** and **Supplemental Figure S1D**). Centrocytes are defined as CXCR4^-^ B_GC_-cells; they belong to the LZ compartment where B cells were also described as CD83^+^ (1, 17). The mean ± standard deviation of CD23-expressing LZ B_GC_-cells was 6.99% ± 1.45 in tonsils and 7.7% ± 3.99 in rLNs (**Figure 1D** and **Supplemental Figure S1E**). In B cells, CD23 expression is induced by Tfh-derived cytokines in general and by IL-4/STAT6 signaling in particular. CD23^+^ and CD23^-^ LZ B_GC_-cells expressed similar levels of IL-21 and IL-4 receptors, CD40 and Ki-67, however, we found a significantly higher proportion of CCR6^+^ cells in the CD23^+^ subset consistent with Duan et al. (24) in mice. These cells could correspond to memory B cell precursors (**Figure 1E** and **Supplemental Figure S1F**) (25).

Given that CD23 expression is signaling-dependent, we hypothesized that CD23^+^ LZ B_GC_-cells correspond to B_GC_-cells that had recently engaged in a productive synapse with cognate Tfh and might therefore harbor a more restricted BCR repertoire than their CD23^-^ counterparts. We assessed common CDR3 clusters and variable (V) gene usage for both IgM and IgG BCRs in CD23^+^ and CD23^-^ LZ BGC-cells isolated from rLNs of three different subjects. We found common CDR3 clusters in all three subjects, with no significant differences in V gene usages between IgMs and IgGs. Interestingly, for one subject we noticed and enrichment for somatic mutations in CDR1, 2 & 3 in CD23^+^ cells compared to CD23^-^ counterparts. No definitive conclusion can be drawn, however, these results showed that CD23^+^ and CD23^-^ LZ B_GC_-cells have very similar repertoires and so probably share a common initial activation pathway (**Supplemental Figure S1G**).

### An Analysis of *FCER2/*CD23 Expression Reveals Heterogeneities in LZ B_GC_-Cells

**Figure 2:**
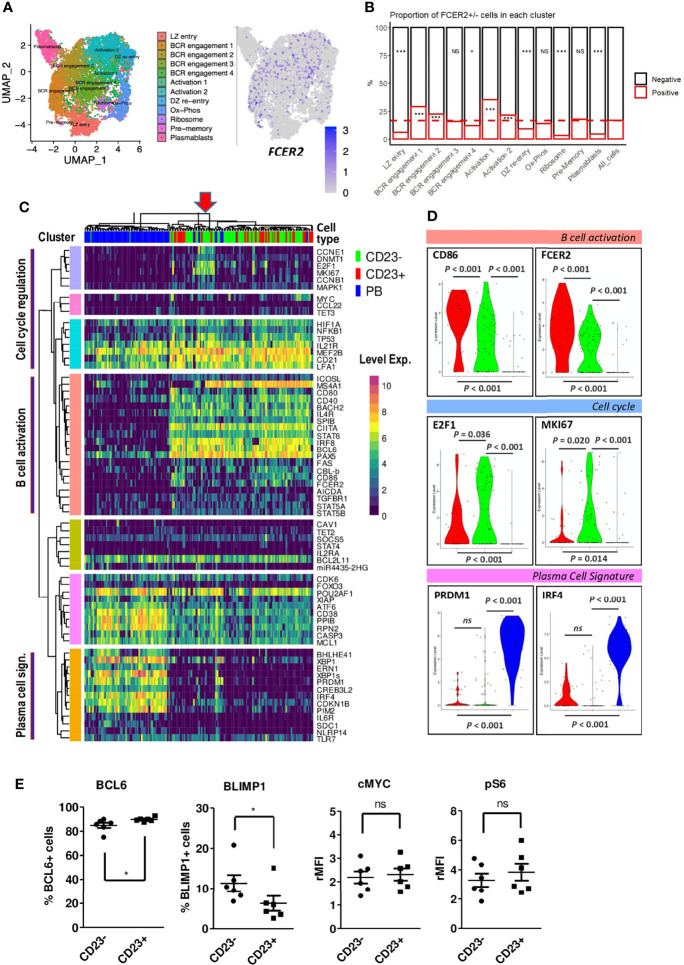
*FCER2/*CD23 expression delineates specific LZ B_GC_-cell subsets. **(A, B)** Single-cell RNA-seq data for human tonsil-derived CXCR4^lo^CD83^hi^ LZ B_GC_-cells in the G0-G1 phase of the cell cycle, from Holmes et al., 2020. **(A)** UMAP of the 12 specific clusters defined by Holmes et al. and expression of *FCER2* in these clusters. **(B)** Distribution of *FCER2* ^+^ (red) and *FCER2* ^-^ (black) non-proliferating LZ B_GC_-cells, in the 12 clusters. Dotted line represent the mean value of positive cells for all clusters (last barplot named All_cells) (Chi2 test for each population vs. total cells (*P < 0.05, ***P < 0.001; significant test are indicated in the barplot of enriched CD23-negative or -positive populations accordingly). **(C)** Selected view of the heatmap with unsupervised clustering of single-cell RT-qPCR data comparing paired cell-sorted CD23^+^ and CD23^-^ LZ B_GC_-cells (CD19^+^IgD^-^CD10^+^CD38^+^CXCR4^lo^) and PBs (CD19^+^IgD^-^CD38^bright^). Annotation of three clusters of genes linked to specific cell functions identified with GeneMANIA related to PCs, B cell activation and cell proliferation. The red arrow on the top of the heatmap indicate in cluster of cycling cells, mostly CD23^-^ LZ B_GC_-cells, that are positive for *MYC* expression represented in the Y-axis pink cluster. **(D)** Violin plot comparisons of the expression of few selected genes in CD23^+^, CD23^-^ LZ B_GC_-cells and PBs. **(E)** Flow cytometry analysis of BCL6, BLIMP1, c-MYC and pS6 in tonsil CD23^+^ and CD23^-^ LZ B_GC_-cells. Results are expressed as the percentage of positive cells or the relative mean fluorescence intensity (rMFI) (*P ≤ .05; "ns" for non significant; Mann-Whitney test).

In humans, the CD23 protein is encoded by the *FCER2* gene. To detect possible transcriptional differences between LZ B_GC_-cells as a function of *FCER2* expression, we compared previously published single-cell (sc) RNA-seq data obtained from tonsil-derived B_GC_-cells (26). A comparison of 8,465 DZ cells and 11,118 LZ B_GC_-cells revealed a highly significant difference between the proportions of *FCER2*-expressing cells (2.9% and 15.3%, respectively) (**Supplemental Figure S2A** *, Left panel*). Among the 11,118 LZ B_GC_-cells, 2,360 (21%) had a proliferative signature (i.e., S-G2-M genes expression) and among them, only 231 (9.8%) cells (primarily in the S phase cluster) expressed *FCER2* (**Supplemental Figure S2A** *, Right panel*). To investigate the heterogeneity of *FCER2* expression in the LZ compartment and follow the same rationale as Holmes et al. (26), we focused our analysis on 8,758 nonproliferating LZ B_GC_-cells (i.e., those in the G0-G1 stage of the cell cycle) which presented 1,465 (16.8%) cells expressing *FCER2* (**Figures 2A, B**). The uniform manifold approximation and projection (UMAP) representation of the original 12 specific clusters allowed to detect the projection of *FCER2-*positive cells and revealed that this gene expression was significantly associated with cell activation clusters and two BCR engagement clusters. In contrast, five clusters presented significantly weaker expression; they included the PB signature, two clusters associated with B_GC_-cell transition between the DZ and the LZ, and one Ribosome cluster (**Figure 2B**).

Taken as a whole, these data showed that the majority of *FCER2^+^* cells belong to a small number of activated, non-proliferating LZ B_GC_-cells that might have been diverted from a PC fate. In contrast, *FCER2*-negative LZ B_GC_-cells were more heterogeneous in their distribution and presented a relative prevalence in the PB subset by taking into account the significant low number of *FCER2*-positive cells in this compartment. In mice, it has been suggested that most pre-plasmablasts are derived from positively selected cells i.e., expressing *MYC* gene (13, 20), we found among the 1,518 human MYC-positive LZ B_GC_-cells from Holmes et al. (26) dataset that 316 (21%) and 1,202 (79%) were, respectively, positive and negative for *FCER2* expression. This result was consistent with data obtained recently in mice (24). Altogether, these data suggest that *FCER2*-negative LZ B_GC_-cells were either unable to express *FCER2* or had not received sufficient Tfh cell help to express it. In particular, *FCER2*-negative LZ B_GC_-cells might include activated B cells that have been committed to PB differentiation and therefore have switched off the IL-4/STAT6 pathway, thereby preventing the expression of *FCER2/*CD23 (19).

In order to complete this *in silico* analysis we explored the heterogeneity of the LZ B_GC_-cell compartment by comparing CD23-negative and CD23-positive cells freshly sorted from tonsils, and CD19^+^IgD^-^CD38^bright^ PBs were included in the analysis (gating strategy, in **Supplemental Figure 2B**). We applied a sensitive sc-qRT-PCR approach to profile the expression of a set of selected genes from our previous study (**Supplemental Material, Method & Tables, Table 2**) (19). An unsupervised clustering analysis revealed three gene clusters linked to specific cell functions identified with GeneMANIA (https://genemania.org/): cell cycle regulation, B cell activation and PC signature (**Figure 2C**). Only a few genes in each cluster were significantly differentially expressed between CD23^+^ and CD23^-^ cells, including *FCER2* and B cell activation markers *CD86* and *NFKB1* (**Supplemental, Material, Method & Tables, Table 6** *).* Note that *PRDM1* and *IRF4* genes linked to PC differentiation were not differentially expressed (**Figure 2D**). However, CD23^-^ compared to CD23^+^ LZ B_GC_-cells expressed higher levels of cell proliferation *E2F1* and *MKI67* genes (**Figure 2D**). The cell proliferation signature was primarily attributed to a group of CD23^-^ LZ B_GC_-cells that also expressed *MYC* (**Figure 2C**). To complete our exploration on a higher number of analyzed cells, differentiation-associated transcription factors assessed by flow cytometry showed a significant enrichment in BCL6-negative and Blimp1-positive cells in CD23^-^ LZ B_GC_-cells. In contrast, both subsets expressed similar levels of c-MYC and phospho-p70 S6 kinase (pS6), two markers related to B-cell selection (**Figure 2E**) (7, 8, 27). Altogether, these findings suggest that the CD23^-^ population comprises B cells committed to PC differentiation.

### CD23 Expression of LZ B_GC_-Cells Depends on Response to Tfh-Driven Stimulation

**Figure 3:**
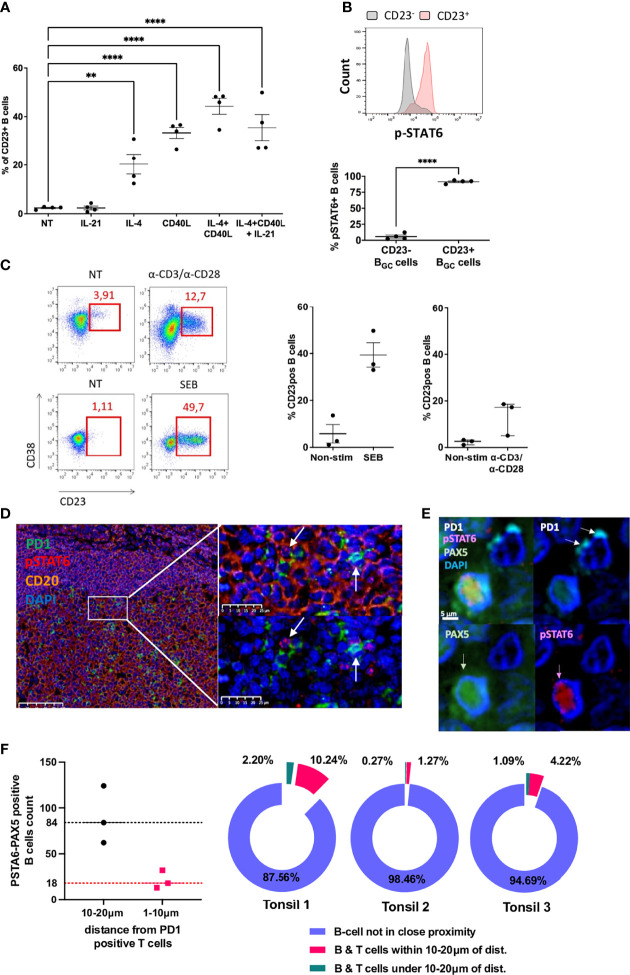
CD23 expression of LZ B_GC_-cells depends on response to Tfh-driven stimulation. **(A)** Flow cytometry analysis of CD23^+^ LZ B_GC_-cells obtained after 12 h of culture with IL-4, CD40L and IL-21 alone or in combinations (**P < .01; ****P < .0001; one-way multiple comparisons test). **(B)** Flow cytometry analysis of pSTAT6 induction on post-stimulation CD23^+^ and CD23^-^ LZ B_GC_-cells, in four independent experiments: Upper panel, a representative flow histogram; Lower panel, quantitative and statistically significant analysis. **(C)** LZ B_GC_-cells and Tfh cells were co-cultured without treatment (NT) or with aCD3/aCD28 or staphylococcal enterotoxin B (SEB) protein for 24 h and subsequently analyzed for CD23 and CD38 expression, using flow cytometry: left panel, representative flow graphs; right panel, results of three independent experiments. **(D)** GC immunohistostaining on paraffin-embedded tonsil sections with DAPI (blue), CD20 (yellow), PD1 (green), and pSTAT6 (red) presenting two high magnification views, showing pSTAT6^+^ B cells in the proximity of PD1^+^ Tfh cells (white arrows). **(E)** An image compatible with a cognate B cell-Tfh cell contact characterized by pSTAT6^+^ B cell in contact with PD1^+^ Tfh cell; immunohistostaining on paraffin-embedded tonsil sections with DAPI (blue), PAX5 (green), PD1 (white), and pSTAT6 (red). **(F)** Number of pSTAT6-PAX5 positive cells in three tonsils in the vicinity of PD1-positive T cells (left panel) and proportion of B cells closed to T cells among total of pSTAT6/PAX5-positive cells (right panel) in each tonsil.

To study the effect of Tfh cell help on the membrane expression of CD23 on LZ B_GC_-cells, we first cultured LZ B_GC_-cells for 12 h with IL-4, CD40, IL-21 or combinations of these. IL-4 alone, CD40 alone and especially a combination of IL-4 and CD40 were effective in inducing the significant upregulation of CD23 expression (**Figure 3A**). Despite similar expression levels of IL-4R, CD40 and IL-21R on post-stimulated CD23^+^ and CD23^-^ LZ B_GC_-cells, pSTAT6 was only induced in CD23^+^ B cells, and chemical inhibition of pSTAT6 blocked the CD23 expression (**Figure 3B** and **Supplemental Figures S3A, B**). Since the production of IL-21 and IL-4 by Tfh cells predominantly supports B_GC_-cells (along with CD40L) (28, 29), we used these three stimuli in our subsequent experiments on primary LZ B_GC_-cells. The data were reproducible, up to 50% of the cells were CD23^+^ after 12 h of culture, cell viability was over 70% (data not shown) and cell proliferation was similar in CD23^+^ and CD23^-^ cells (**Supplemental Figure S3C**). We next co-cultured LZ B_GC_-cells with paired autologous CD4^+^CXCR5^+^ICOS^+^PD1^+^ Tfh cells for 24 h and then analyzed CD23 expression in three independent experiments. Only a weak increase in CD23 expression was observed. In contrast, when Tfh cells were activated either with α-CDA/α-CD28 antibodies or staphylococcal enterotoxin B protein, the CD23 expression increased markedly; 13.6+/-7.4% and 39.4+/-9.0% of the B cells were CD23^+^, respectively (**Figure 3C**).

Taken as a whole, our present data suggest that the low number of CD23^+^ LZ B_GC_-cells is linked to a lack of effective, complete Tfh cell help in GCs - a key limiting resource for which the B cells compete (29). After early B cell-Tfh cell contact and in the absence of sustained Tfh support, CD23^+^ B_GC_-cells might also undergo apoptosis; this has been described *in vivo* for B_GC_-cells after 24 h of antigen recognition and *in vitro* for CD23^+^ post-activated B cells (19, 30). We confirmed the latter observation and showed that only CD23^+^ LZ B_GC_-cells were more numerous when the pan-caspase inhibitor QVD-OPH was added to the culture for 12 h (**Supplemental Figure S3D**).

Since IL-4 signaling goes through the STAT6 pathway, we next determined the number of B cells receiving Tfh-derived IL-4 in a contact dependent manner at a given point in time. To this end, we looked for nuclear expression of pSTAT6 in CD20^+^ B_GC_-cells by staining tonsil tissue sections. Tfh cells were detected by staining for PD1. By using a machine learning approach to automatically detect cells on microscopy images, we estimated that approximately 2 to 3% of B_GC_-cells were pSTAT6^+^CD20^+^ (**Figure 3D** and **Supplemental Figure S3E**). By replacing CD20 with PAX5, we could estimate the number of pSTAT6^+^ cells in the vicinity of PD1^+^ T cells for three tonsils. Overall, among the average of 2880 PAX5^+^/pSTAT6^+^ B cells per tonsil, we found 18 (min 13- max 32) and 84 (min 62- max 124) of these cells located, respectively, within 10μm and 20μm of PD1^+^ T cells. This corresponds to an average of 1.2% and 5.2% of B cells in less than 10µm or within 20µm proximity of T cells that may sufficient to favor contact between cognate B and T cells (**Figures 3E, F**). For the reasons mentioned above, we could not use the CD23 marker in parallel.

### CD23^-^ Activated LZ B_GC_-Cells Contain PC Precursors

**Figure 4:**
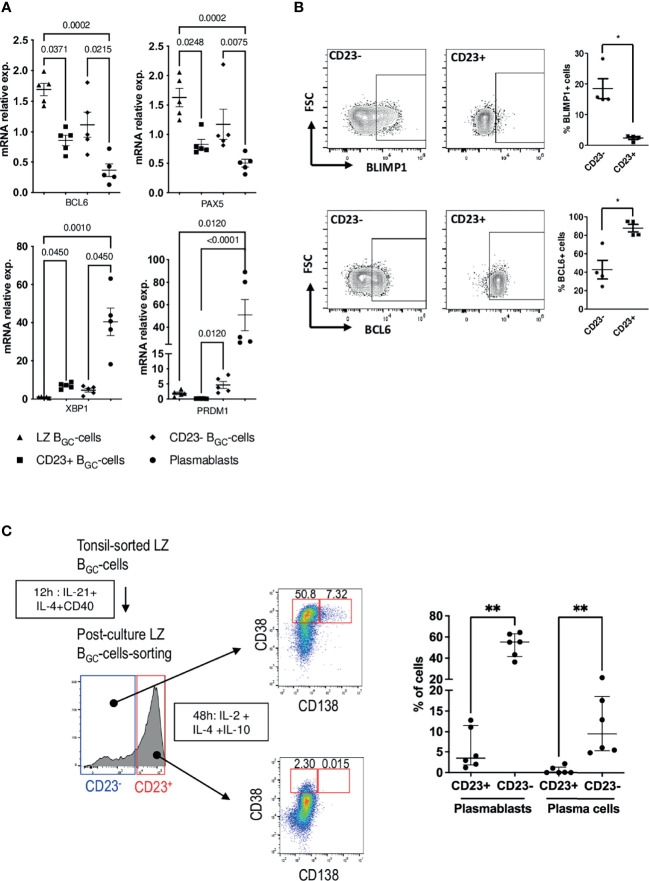
CD23^-^ LZ B_GC_-cells contain PB precursors. **(A)** *BCL6*, *PAX5*, *XBP1* and *PRDM1* gene expression after 12 h culture in the presence of IL-4, CD40L and IL-21 for LZ B_GC_-cells *vs*. freshly sorted, paired LZ B_GC_-cells and PBs (one-way Anova & Kruskal-Wallis multiple comparisons test). **(B)** Flow cytometry analysis of BLIMP1 and BCL6 expression in post-culture CD23+ and CD23- LZ B_GC_-cells: Left panel, representative flow graphs; Right panel, results of four independent experiments (*P ≤ .05; Mann-Whitney test. **(C)** Post-culture CD23^+^ and CD23^-^ LZ B_GC_-cells were subsequently maintained for 48 h in the presence of IL-2, IL-4 and IL-10 prior to flow cytometry analysis, in order to detect CD38^bright^CD138^-^ PBs and CD38^bright^CD138^+^ PCs. Left panel, graphical representation of the experiment and right panel, detected percentages of PBs and PCs for six independent experiments (**P ≤ .01; Mann-Whitney test).

Since the LZ B_GC_-cell compartment was heterogeneous with regard of CD23 expression after stimulation, we decided to study CD23^-^ and CD23^+^ LZ B_GC_-cells obtained after 12 h of culture in the combined presence of IL-21, IL-4 and CD40L (hereafter referred to as pcCD23^-^ or pcCD23^+^ LZ B_GC_-cells, where “pc” indicates “post-culture”). The gating strategy for cell sorting is presented in **Supplemental Figure S4A**. A comparison of these two subsets showed that *FCER2* expression was elevated only in pcCD23^+^ LZ B_GC_-cells (**Supplemental Figure S4B**). With regard to the four characteristic transcription factors involved in PC differentiation, there were no differences between the subsets in *BCL6, PAX5* and *XBP1* expression but *PRDM1* was significantly upregulated in pcCD23^-^ LZ B_GC_-cells (**Figure 4A**). Flow cytometry analysis showed significantly higher BCL6 expressing cells in the CD23^+^ subset while the CD23^-^ counterpart contained more BLIMP1^+^ cells (**Figure 4B**).

We then evaluated the subsets’ ability to differentiate into PBs (CD38^bright^) and PCs (CD138^+^). To that end, pcCD23^-^ and pcCD23^+^ LZ B_GC_-cells were cultured separately for 48 h in the presence of IL-2, IL-4 and IL-10. Statistically significant results showed that only pcCD23^-^ LZ B_GC_-cells were able to give rise to a significant number of CD38^bright^ and CD138^+^ differentiated cells (**Figure 4C**).

The stimulation experiments were performed on total LZ B_GC_-cells. However, to further specifically explore the CD23^+^ minority compartment of LZ B_GC_-cells, sorted tonsillar CD23^+^ LZ B_GC_-cells (**Supplemental Figure S2B**) were cultured for 24 h under the same conditions as above and analyzed by sc-qRT-PCR. No marker related to PC differentiation was detected after stimulation (**Supplemental Figure S4C**).

### Activated CD23^-^ LZ B_GC_-cells Follow a Continuous, Homogeneous Trajectory Towards PBs

**Figure 5:**
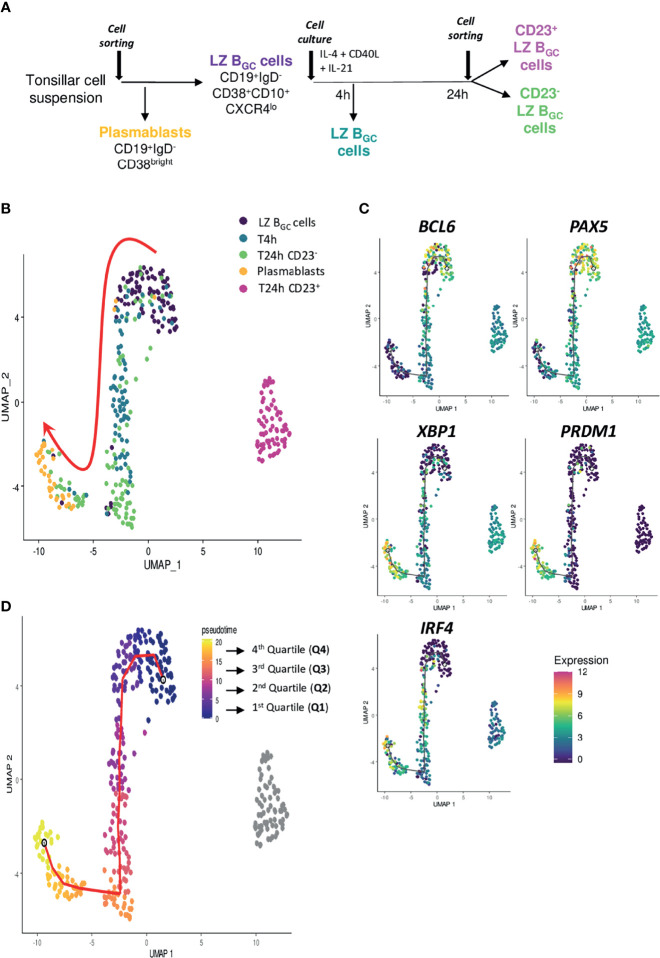
Cell destiny of human LZ B_GC_-cells after Tfh-like stimulation. **(A)** The experimental plan, with five different cell populations (colored script) analyzed using sc-RTqPCR. **(B)** The UMAP representation (colored according to the experimental conditions) highlighted two different fates as a function of CD23 expression. **(C)** Projection of B cell identity genes *(BCL6* and *PAX5*) and PC identity genes (*PRDM1*, *XBP1*, and *IRF4*) onto the UMAP representation. **(D)** The UMAP representation (colored according to the Monocle pseudotime), showing an ordered, progression in four quadrants from LZ B_GC_-cells to PBs.

Our *in vitro* results and the literature data on LZ B_GC_-cells (19) confirmed that the CD23^+^ and CD23^-^ subsets contained cells with distinct cell fates. To further explore this difference, we tracked spatiotemporally gene expression in stimulated LZ B_GC_-cells. To this end, selected genes (**Supplemental Material, Method & Tables, Table 2**) (19) were analyzed using sc-qRT-PCR at different time points of the culture. Thus, tonsil sorted PBs were compared with paired LZ B_GC_-cells after 4 h and 24 h of culture with IL-21, IL-4 and CD40L. For the 24 h time point, CD23^+^ and CD23^-^ cells were sorted (**Figure 5A**). To investigate the time course of changes in cell populations, we applied the Monocle trajectory inference algorithm (https://doi.org/10.1038/nbt.2859). UMAP reduction of the five cell populations highlighted two clusters that differed in their cell fate as a function of CD23 expression (**Supplemental Figure S5**). Only 24 h CD23^-^ LZ B_GC_-cells followed continuous, homogeneous trajectory *via* expression of the *BCL6, PAX5, IRF4, XBP1* and *PRDM1* genes associated with differentiation into PBs (**Figures 5B, C**). However, some LZ B_GC_-cells had started to migrate along the differentiation trajectory as early as 4 h, and clustered with PBs. Using Monocle, the UMAP projection was color-coded according to the pseudo-time; this showed a well-ordered progression in quadrants from LZ B_GC_-cells to PBs (**Figure 5D**).

### The Time Course of Gene Expression Changes During the Differentiation of CD23^-^ LZ B_GC_-Cells

**Figure 6:**
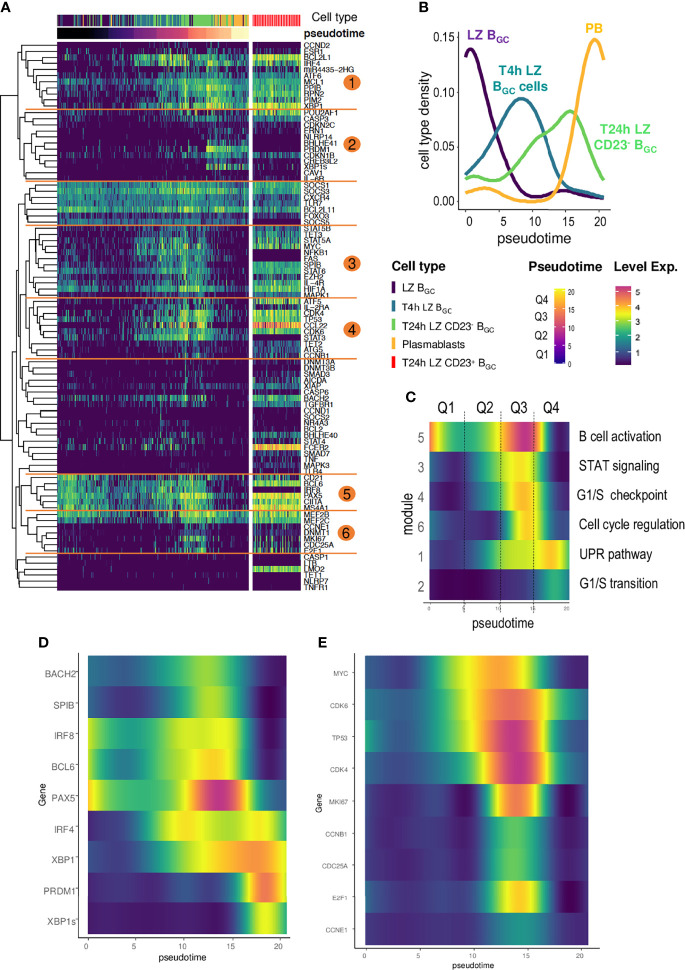
The time course of gene analysis during LZ B_GC_-cell differentiation. **(A)** A heatmap computed for Monocle-ordered cells (LZ B_GC_-cells, PBs, T4h LZ B_GC_-cells, T24h CD23^+^ LZ B_GC_-cells, and CD23^-^ LZ B_GC_-cells), showing six different gene modules annotated with GeneMANIA. The pseudotime scale was calculated from the gene expression data. **(B)** Density of cell subsets in each quadrant of the pseudotime. **(C)** Smooth analysis of the time course of each module by pseudotime quadrant (Q1 to Q4) for all cell subsets except T24h CD23^+^ LZ B_GC_-cells. **(D)** The time course of gene expression during B cell activation and differentiation into PCs showing (i) a bimodal expression of *IRF4*, (ii) a striking peak for *PAX5*, (iii) *PRDM1* elevation synchronized with *PAX5* decline, and (iv) the specific expression of *XBP1s* in the last quadrant of the pseudotime compared to *XBP1*. **(E)** Gene expression during the differentiation of CD23^-^ LZ B_GC_-cells for Monocle-ordered cells showing a sequential transition from LZ B_GC_-cells to PBs for *MYC* expression, MYC-target genes and gene involved in cell cycle re-entry.

The expression heatmap for Monocle-ordered cells showed a sequential transition from CD19^+^IgD^-^CD38^+^CD10^+^CXCR4^-^ LZ B_GC_-cells to CD19^+^IgD^-^CD38^bright^ PBs (**Figure 6A**). This situation enabled us to analyze changes over time in gene expression from one quadrant to the next and excluded T24h CD23^+^ B_GC_ cells in agreement with the above result (**Supplemental Figure S5**). Each quadrant is enriched with a specific subset of cells (**Figure 6B**). The computed clustering allowed to identify six gene modules annotated by GeneMANIA (**Figure 6C**) characterized by a specific expression time course and linked to transcription factors, cell identity factors, and cell functions (**Supplemental Material, Method & Tables, Table 5**). Modules 1 and 2 were linked to PC identity genes. Modules 3, 5 & 6 were linked to a B cell activation state. In fact, Module 3 encompassed several transcription factors (including MYC) and preceded Module 6, which was linked to transient cell cycle entry prior to extinction in the fourth quadrant (Q4). In Module 4, a transient expression pattern peaked during Q3 - probably in response to upstream factors such as MYC. It included the expression of *ATF5*, a transcription factor involved in the CREB3L2-ATF5-MCL1 survival pathway and which acts as a stress sensor (31). Module 5 featured a transient decline in the expression of B cell identity genes in Q1, a sharp increase in Q2 and Q3, and a drastic fall in Q4.

Our model of LZ B_GC_-cell differentiation as a function of CD23 expression makes it possible to calculate how levels of transcription factors change in space and over time. The changes over time observed here were in line with the literature data (**Figure 6D**) (32). The *IRF4* gene was expressed in two phases, with an initial peak preceding the increase in expression of the B cell identity genes *PAX5, BCL6, BACH2*, and *SPIB*. The second peak occurred when the four factors were no longer expressed. *PAX5* expression showed a striking increase and peaked at the end of Q3 before dropping sharply and thus de-repressing the expression of *PRDM1*. This result is consistent with the fact that LZ B_GC_-cells enter an activation state before they switch to the PB differentiation pathway (32). Although *PRDM1* and *XBP1s* are late transcription factors that seal the commitment to PBs, *XBP1* expression starts as soon as B cells activate. Furthermore, our model highlights the MYC imprint involved in the differentiation of B cells; our results are in agreement with MYC’s description as a mediator of B_GC_-cell survival and cell-cycle re-entry and as a marker of positive selection (7, 32, 33, 34). *MYC* expression is absent in sorted LZ B_GC_-cells but rises quickly in the timeline followed by the expression of early MYC-target genes *TP53*, *CDK4* and *CDK6* (**Figure 6E**) (35, 36) and then the proliferative genes from Module 6 plus *CCNB1* (Module 4) – marking cell cycle re-entry.

### Overview of IL-4 Signature Integration in LZ B_GC_-Cells

**Figure 7:**
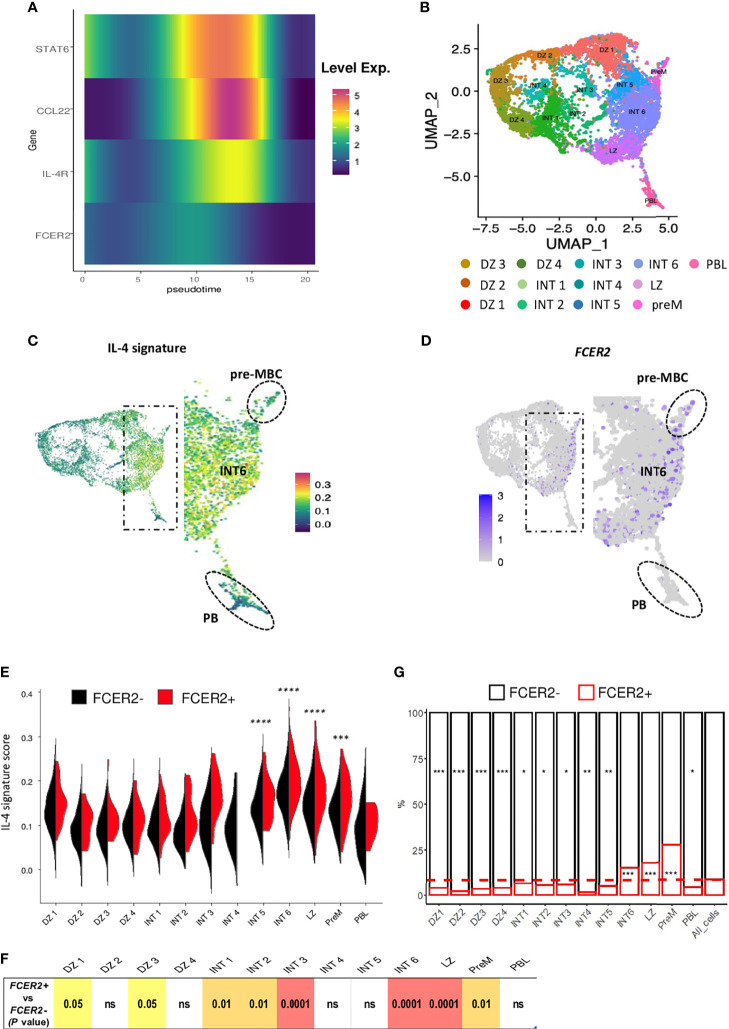
Overview of the IL-4 signature in LZ B_GC_-cells. **(A)** The time course of expression of IL-4-induced genes by quadrant showing a difference between the almost flat *FCER2* expression and the strong *CCL22* expression. **(B–G)** Single-cell RNA-seq data for human tonsil-derived total B_GC_-cells, from Holmes et al., 2020**. (B)** UMAP representation of the 13 specific clusters defined by Holmes et al. ***(*C*)*** UMAP showing cell expression of the IL-4 signature, noteworthy that positive cells are located in the right part of the map mainly in intermediate 6 (INT6) cluster (enlarged view) with an expression maintained in pre-MBCs (Top, dotted circle) while PBs are negative (Bottom, dotted circle). **(D)** UMAP showing *FCER2* ^+^ cells; the enlarged view shows the preferential enrichment of these cells on the far right of the map depicting a ridge line extending from the INT6 cluster to the pre-MBC cluster (top, dotted line circle). **(E)** Violin plots comparing IL-4 signature expression in each of the 13 GC B cell clusters for *FCER2^+^* (red) and *FCER2^-^* (black) cells (Pairwise t-test for IL-4 signature comparison between clusters is indicated above the plot; ***P < .001; ****P < .0001). **(F)** Table of *P* values of Wilcox test comparing *FCER2^+^* and *FCER2^-^* B_GC_-cells for IL-4 signature in each of the 13 clusters. **(G)** Percentage of *FCER2^+^* and *FCER2^-^* B_GC_-cells in the 13 clusters. Dotted line represent the mean value of positive cells for all clusters (last barplot named All_cells). Chi2 test for each population compared to total cells (*p < 0.05, **p < 0.01, ***p < 0.001, "ns" for non significant); significant test are indicated in the barplot of enriched FCER2 neg or pos populations accordingly.

The pseudo-time inferred from the Monocle algorithm provided an overview of the signal delivered by IL-4 (present in our differentiation cocktail) through the expression of *STAT6, IL4R, FCER2* and the well-established IL-4/STAT6 target *CCL22* (37) (**Figure 7A**). In line with our previous results (19), LZ B_GC_-cells are unable to upregulate *FCER2* once they have committed to the PBs pathway but do produce a clear *STAT6* response marked by the expression of *IL4R* and *CCL22*. We then positioned on the sc-RNA-seq UMAP plot of total B_GC_-cells from Holmes et al. (26) genes a B cell-specific IL-4 response signature (**Figure 7B**) (38). Positive cells for this signature were predominantly located in an area between pre-MBC and PB clusters, the latter two representing the two distinct fates for B_GC_-cells (**Figure 7C**). A similar distribution was obtained for cells expressing *FCER2* with higher density on the far right of the map, drawing a ridge line extending from the intermediate (INT) 6 cluster to the pre-MBC cluster (**Figure 7D**). Statistical analysis showed a striking enrichment for the IL-4 signature in INT5, INT6, light-zone (LZ) and pre-memory clusters compared to other clusters (**Figure 7E**). IL-4 signaling was significantly enriched in *FCER2^+^* cells compared to *FCER2^-^* counterparts in some clusters including INT6, LZ and pre-memory (**Figure 7F**). Interestingly, these three latter also exhibited the highest proportion of *FCER2^+^* cells compared to other clusters (**Figure 7G**). Finally, unlike pre-MBCs, most differentiated PBs were negative for both IL-4 signature and *FCER2* expression, confirming that in last steps of the PB commitment, cells turned off their IL-4/STAT6/FCER2 signaling (19).

## Discussion

In the present study, we investigated the functional diversity of human LZ B_GC_-cells collected at a single point in time from tonsils and rLNs. We found that the PC precursors are contained within the CD23-negative LZ B_GC_-cell compartment. Based on our findings, time course gene expression analysis provided new information on the LZ B_GC_-cells’ commitment to differentiation and, in particular, on the kinetics and interconnexion between transcription factors.

Conventionally, it has been thought that like in mice, human B_GC_-cells express CD23 in response to molecules produced by Tfh cells (19). Our results show that less than 10% of tonsil B_GC_-cells expressed this protein, this number increasing when considering only LZ B_GC_-cells. In contrast, in rLNs, LZ B_GC_-cells express much higher levels of CD23 positive cells reflecting likely that unlike chronically inflamed tonsils, rLN tissues are produced in an acute response to a more limited number of antigens. In addition, our study confirmed previous data by showing that LZ stromal FDCs express very high levels of the CD23 receptor (22, 23). Complementing very recently published data from Cyster’s team showing that FDC restricted IL-4 availability in the GC (24) and our work revealing the expression of IL-4 receptor and CD40 by FDCs (39), it is tempting to speculate that FDCs activate CD23 expression by trapping molecules produced by Tfh cells in the LZ and then titrating them. In such a case, the LZ B_GC_-cells require effective, lasting contact with Tfh cells for activation leading notably in B cells by the recruitment of the IL-4/STAT6 signaling and CD23 expression (19). Our pSTAT6 protein staining is consistent with the detection of the CD23 marker and the single-cell data on *FCER2* expression; these findings indicate that only a small proportion of LZ B_GC_-cells are engaged in B cell-Tfh cell contacts at a given point in time. These results also agree with (i) the transient, dynamic interactions between B_GC_-cells and Tfh cells observed in GCs (providing opportunities for competition between B cells) and (ii) the fact that up to half of the B_GC_-cells undergo apoptosis every 6 hours due to the absence of active positive selection and irrespective of the BCR affinity (34). *In silico* reanalyzed data from Holmes et al. (26) shows that *FCER2*-expressing LZ B_GC_-cells are activated, BCR^+^ cells that might upregulate *CD86* - a gene whose expression in MBCs is induced by IL-21 (40). In addition, flow cytometry detects significantly more CCR6^+^ cells in CD23^+^ than CD23^-^ LZ B_GC_-cells. The sc-qRT-PCR experiment shows no upregulation of genes related to PC differentiation after stimulation of sorted CD23^+^ LZ B_GC_-cells as well as absence of cells downregulating the *FCER2* expression. Overall, our findings support the hypothesis whereby CD23 expression characterizes Tfh-instructed, post-activated LZ B_GC_-cells diverted from a PC fate (40, 41). However, some activated LZ B_GC_-cells may decrease the density of the membrane-bound form of CD23 – a probable reason for the expression of the *FCER2* gene in some CD23^-^ tonsillar LZ B_GC_-cells sorted by flow cytometry – after release as a freely soluble molecule due to ADAM10 sheddase (42, 43). This point may contribute to our BCR repertoire results where CD23^+^ and CD23^-^ subsets are clonally related, which do not exclude that clonal evolution could possibly stand at different stages of their differentiation. Collectively, our data demonstrate that CD23^-^ LZ B_GC_-cells are heterogeneous and contain both stimulated and unstimulated B cells as well as B cells having committed to the PB pathway. In these committed B cells, the IL-4 signal is removed and the *FCER2* gene can no longer be upregulated, the PBs being totally negative for this signaling and this marker. Interestingly, committed CD23^-^ LZ B_GC_-cells maintain a transient ability to upregulate IL-4/pSTAT6-dependent *CCL22* after stimulation; this observation is consistent with the finding that CCL22 promotes positive selection in murine GCs by increasing the chance of productive Tfh help (44). Thus, in the committal step of PB generation, IL-4/STAT6 signaling is definitively repressed before a switch into a PC gene expression pattern during the S phase of the cell cycle (14, 19). In agreement with a recent study, we used a sensitive sc-RT-qPCR to detect proliferating CD23^-^ LZ B_GC_-cells that steadily increased their expression of *MYC* - an indicator of positive selection (45)- after *in vitro* stimulation (20). The computed cell trajectory showed that some LZ B_GC_-cells reached the PB site after just 4 h and indicated that some cells were ready to recycle and differentiate after appropriate stimulation. This finding is consistent with previous data on high-affinity LZ B_GC_-cells that migrate to the DZ and can differentiate into PCs, depending on the amount of CD40 signal captured (13, 46). Overall, the trajectory of CD23^-^ LZ B_GC_-cells (i) sheds light on the time course in gene expression during commitment to the PC pathway, and (ii) enables comparisons with regard to various genes and possible interdependencies. For example, transient expression of IRF4 sustains the expression of *BCL6* and *POU2AF1* (47); *POU2AF1* expression is consistent with its ability to activate B_GC_-cells and induce GC formation by (at least in part) underpinning the IL-4 response (48) and then enhancing the generation of PCs (49).

**Figure 8:**
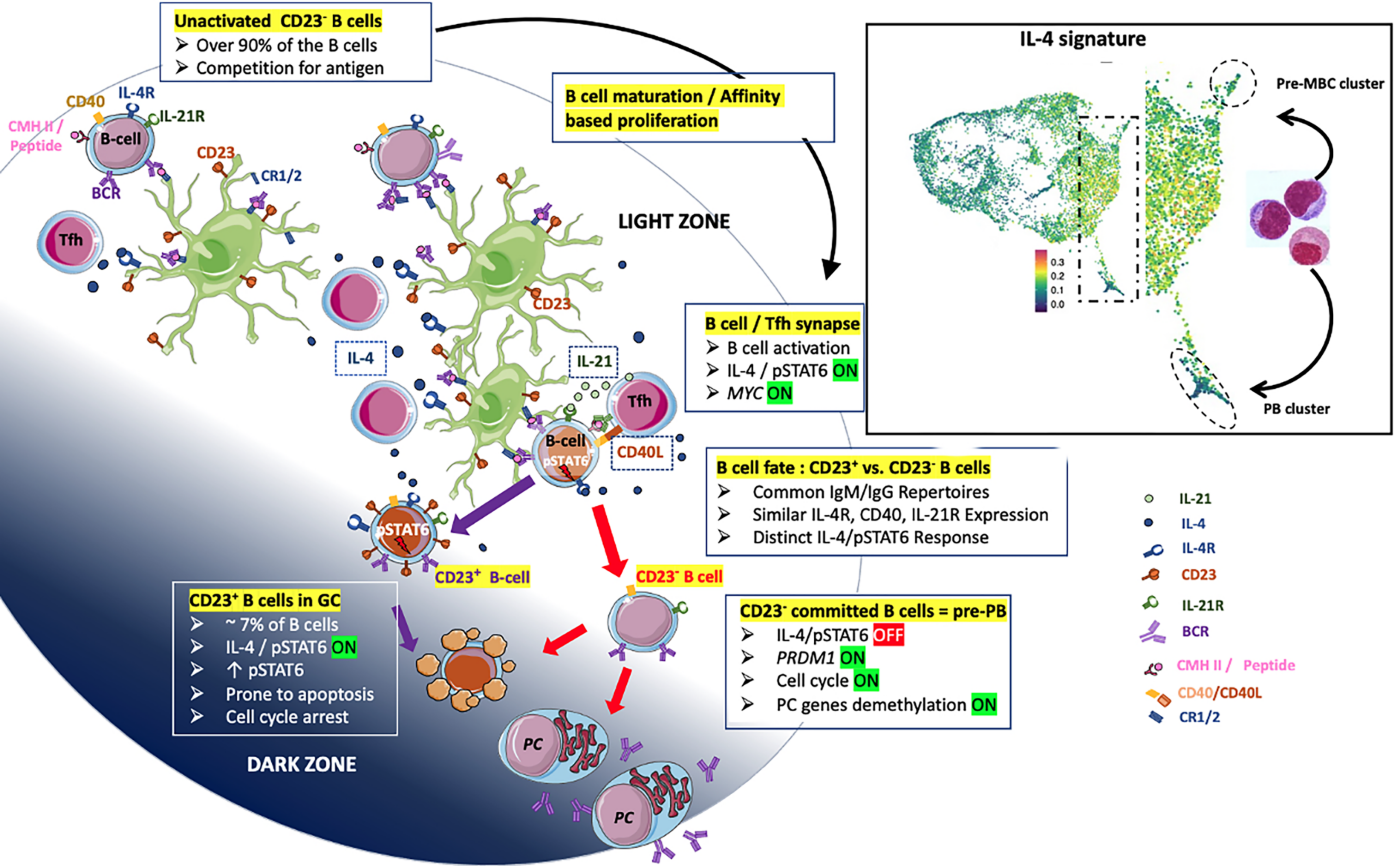
Proposal of an instructive differentiation model of LZ B_GC_-cells integrating the expression of the CD23 marker. B_GC_-cells are predominantly CD23^-^ compete for antigen (Ag) when only limited amount of Ag is available (top-left B cells). Fit cells proliferate, retrieve Ag deposited on follicular dendritic cells (FDCs) and receive survival signals from stromal cells (top-middle part). Note that FDCs express IL-4R and may take available IL-4 cytokine - in a non-directed manner [IL-4 broadcasting as called in (24)] – produced by Tfh. Both, high and low-affinity cells process Ag and present - in proportion to its affinity - peptide-MHC complex which supports the interaction with cognate Tfh and the delivery of crucial molecules including IL-21, CD40L and IL-4 leading to pSTAT6 expression (right-middle part). The split of the B cell fate depends on the integration of sufficient signals which impacts the maintenance or extinction of the IL-4/pSTAT6 signaling pathway. B cells that have quenched the IL-4/STAT6 signal are unable to express the CD23 marker and progress further to the PB axis of differentiation (bottom-right). These CD23^-^ LZ B_GC_-cells correspond to pre-PB described previously (19) which express transiently *MYC* leading to cell cycle and the committal step of differentiation described elsewhere (14). In contrast, CD23^+^ LZ B_GC_-cells which have maintained IL-4/STAT6 signaling are prone to apoptosis or give rise to pre-MBCs; the maintenance of CD23 expression depends on the presence of IL-4 and CD40 stimuli (bottom-middle). In the right square, a view of the **Figure 7C** concerning scRNA-seq data for B_GC_-cells representing the fate of activated LZ B_GC_-cells between pre-MBC or PB outputs.

Overall, the CD23 marker might be of value in answering questions about the differentiation of normal B_GC_-cells. Based on extensive data in the literature including our previously published data (14, 50, 51) and present results complemented by explorations on previous scRNA-seq (26) as well as recent results of Duan et al. (24), we propose an instructive model of the maturation and fate of human B_GC_-cells in LZ (**Figure 8**). The majority of B_GC_-cells are CD23-negative, progress in an affinity-based proliferation and compete for Tfh help leading to a productive IL-4/pSTAT6 response. B cells committed in PB differentiation first lose their capacity to express the CD23 marker, then IL-4/pSTAT6 signaling and finally trigger final PC programming supported by a specific demethylation process (14). In this context, CD23-negative committed B cells correspond to pre-PBs, diverted from cell death, they transiently express *MYC* which triggers the cell cycle and gives rise to a metamorphosis of B cells into PBs. The cell fate split between pre-MBC and PB destinies may take place in the INT6 cluster of B_GC_-cells. There, B cells express the IL-4/STAT6 signature and then migrate either to the pre-MBC group or after their IL-4/STAT6 signaling has been turned off, to the PB cluster. Taken as a whole, the present results have implications for the design of future studies on the differentiation of both normal human B_GC_-cells and their malignant counterparts. Indeed, in patients with follicular lymphoma, the survival was significantly better in patients carrying CD23^+^ FL B cells (52).

## Materials and Methods

### Human Samples and Primary B Cell Purification

Tonsils obtained from children undergoing routine tonsillectomy were obtained following approval by the French Ministry of Higher Education and Research (reference: AC-2014-2315). The children’s parents provided their informed consent to use of the samples for research purposes. The study was performed in accordance with the principles of the Declaration of Helsinki. Tonsil-derived B_GC_-cells were enriched by negative selection *via* magnetic cell separation, using the MojoSort Human Pan B Cell Isolation Kit (Biolegend, San Diego, CA) with the addition of biotinylated IgD antibody (BD Biosciences, San Jose, CA) and the AutoMACS deplete program (Miltenyi Biotech, Bergisch Gladbach, Germany). LZ B_GC_-cells (CD19^+^IgD^-^CD10^+^CD38^+^CXCR4^lo^) and PBs (CD19^+^IgD^-^CD38^bright^) were then sorted using a FACSAria system (BD Biosciences). All antibodies used for flow cytometry are listed in **Supplemental Material, Method & Tables, Table 1**.

### Single-Cell qPCR Experiments

Single-cell experiments were performed using the Fluidigm^®^ C1 ™systems according to manufacturer instructions., Briefly, sorted cells were captured with CI Single-Cell Auto Prep integrated fluidic circuits (IFC) 5-10 µm (Fluidigm; 100-5757). Cells were then lysed, and reversed transcription and pre-amplification (Ambion Single cell-to-Ct Kit; 4458237) was done within the C1 system. Gene expression levels was then assessed by qPCR for selected taqman assays using Taqman Gene expression master mix Life technologies; 4369016) on 96.96 Dynamic Arrays IFC (Fluidigm; BMK-M-96.96) within the Fluidigm BioMark™ HD system. The list of TaqMan assay-on-Demand™ used is provided in **Supplemental Material, Method & Tables, Table 2**.

### Histo-Immunofluorescence Staining

Human tonsils and reactive lymph nodes were embedded in Optimal Cutting Temperature Compound (OCT, Sakura) and conserved at -80°C. Cryostat sections (18 µm thick) were fixed with 4% paraformaldehyde for 15 min at room temperature (RT). Sections were then incubated during 1 h with a blocking solution (PBS, 2% Bovine Serum Albumin, 4% donkey serum and 0.1% saponin) at RT and incubated in a humidified chamber overnight at 4° C with primary antibodies. Sections were washed with PBS 0.1% saponin and incubated with secondary antibodies for 1 h at RT. Finally, tissue sections were mounted with Mowiol (Merck) antifade reagent containing 20µM of SytoxBlue nucleic acid stain (Thermo Fischer) and analyzed by confocal microscopy on a SP8 (Leica Microsystems). ImageJ software (National Institutes of Health) was used for image analysis. The list of primary and secondary antibodies used for immunohistofluorescence is provided in **Supplemental Material, Method & Tables, Tables 3, 4**.

For multiplex fluorescence microscopy and analysis, three FFPE samples of human tonsil were provided by the Pathology department of Rennes. Four-micrometer-thick whole-slide sections, obtained with a microtome (Histocore multicut Leicabiosystems, Nanterre, France) from FFPE tissue, were transferred onto plus-charged slides (VWR international), followed by multiplex immunofluorescence staining with a U DISCOVERY 5 plex immunofluorescence (Roche Diagnostics, Meylan, France). Four sequential rounds of staining were performed each including heat deactivation step, followed by incubation with primary antibody and corresponding HRP secondary antibody. Hence, primary antibodies expressions (as described in **Supplemental Material, Method & Tables, Table 3**) were visualized on the same section. HRP enzyme mediated deposition of the tyramide; coupled to respectively rhodamine, DCC, cyanine-5 and FAM fluorophores species (kits Ventana Medical Systems, Tucson, Arizona) that covalently bound to the tissue at the site of the reaction. After four sequential reactions, sections were counterstained with DAPI and cover slipped using fluoromount (Enzo Life Sciences, Farmingdale, NY, USA). Visualization was performed with the Nanozoomer (Hamamatsu Photonics, Massy, France) equipped with the multicolor fluorescence module.

For the pSTAT6-positive B_GC_-cell percentage assessment, an automated analysis by machine learning with the HALO software was performed. After cell segmentation and nuclear detection pSTAT6, CD20, PAX5 and PD1 threshold intensities were set up. Six GCs in three different tonsils were analyzed. Double CD20 or PAX5 and pSTAT6-positive cells and total CD20- or PAX5-positive cells were quantified and a percentage of positive pSTAT6 B cells was assessed.

### Information About RNA-Seq Datasets Used Throughout the Paper

Bulk RNAseq datasets used to select genes analyzed by sc-qPCR in this study are available in the Gene Expression Omnibus database under accession no. GSE136990.

Single-cell-RNAseq datasets from Holmes et al.’s paper (26) are available in the Gene Expression Omnibus database under accession no. GSE139833. sc-gene expression data are available under accession no. GSE139891.

### Statistical Analysis

Quantitative variables were expressed as the mean ± standard deviation (SD). Statistical analyses were performed with Prism software (version 5, GraphPad Software, San Diego, CA) and R software (version 3.6.0). Statistical significance was assessed using the Mann-Whitney nonparametric U test and a one-way Anova and Kruskal-Wallis multiple comparison test (*P ≤ .05; **P < .01; ***P < .001; ****P < .0001).

## Data Availability Statement

Publicly available datasets were analyzed in this study. These data can be found here: 1) The dark zone and light zone bulk single-cell RNAseq gene expression data are available in the Gene Expression Omnibus database under accession no. GSE139833. sc-gene expression data are available under accession no. GSE139891; 2) CD23-positive and CD23-negative RNA-seq data have been deposited in the Gene Expression Omnibus: GSE136990.

## Ethics Statement

Ethical review and approval was not required for the study on human participants in accordance with the local legislation and institutional requirements. The ethics committee waived the requirement of written informed consent for participation.

## Author Contributions

KS performed experiments, designed some experiments, analyzed data. FD analyzed and integrated omics data, created tools for analysis, supervised KS for some experiments and helped draft the manuscript. GC performed and designed experiments, reviewed the paper. SL analyzed single-cell transcriptome data., MH, CD, and AP performed experiments to characterize CD23 marker expression on the various B cell populations. FC analyzed and integrated omics data and created tools for analysis. DR and MC analyzed B cell repertoires. CM performed single-cell experiments. MS and CL performed histology and immunofluorescence experiments. KT analyzed stromal cell staining and B cell-Tfh cell cocultures. TF designed the study, performed transcriptome analysis, supervised the project and wrote the manuscript. All authors read and approved the manuscript.

## Funding

This research was funded by an internal grant from the Hematology Laboratory (Pôle de Biologie, CHU de Rennes, Rennes, France) and two grants from the *Association pour la Recherche contre le Cancer* (ARC, PJA 20181207839 and PJA3 2020060002221).

## Conflict of Interest

The authors declare that the research was conducted in the absence of any commercial or financial relationships that could be construed as a potential conflict of interest.

## Publisher’s Note

All claims expressed in this article are solely those of the authors and do not necessarily represent those of their affiliated organizations, or those of the publisher, the editors and the reviewers. Any product that may be evaluated in this article, or claim that may be made by its manufacturer, is not guaranteed or endorsed by the publisher.
