# Supplementary material for: Committed Human CD23-Negative Light-Zone Germinal Center B Cells Delineate Transcriptional Program Supporting Plasma Cell Differentiation
Source: Front Immunol. 2021 Dec 2;12:744573. doi: 10.3389/fimmu.2021.744573 (PMC8674954; doi:10.3389/fimmu.2021.744573)
Supplement: Supplementary file 1 [file DataSheet_1.zip › Supplemental_Figures_Santamaria-et-al_revised-FINAL.pptx]

## Slide 1
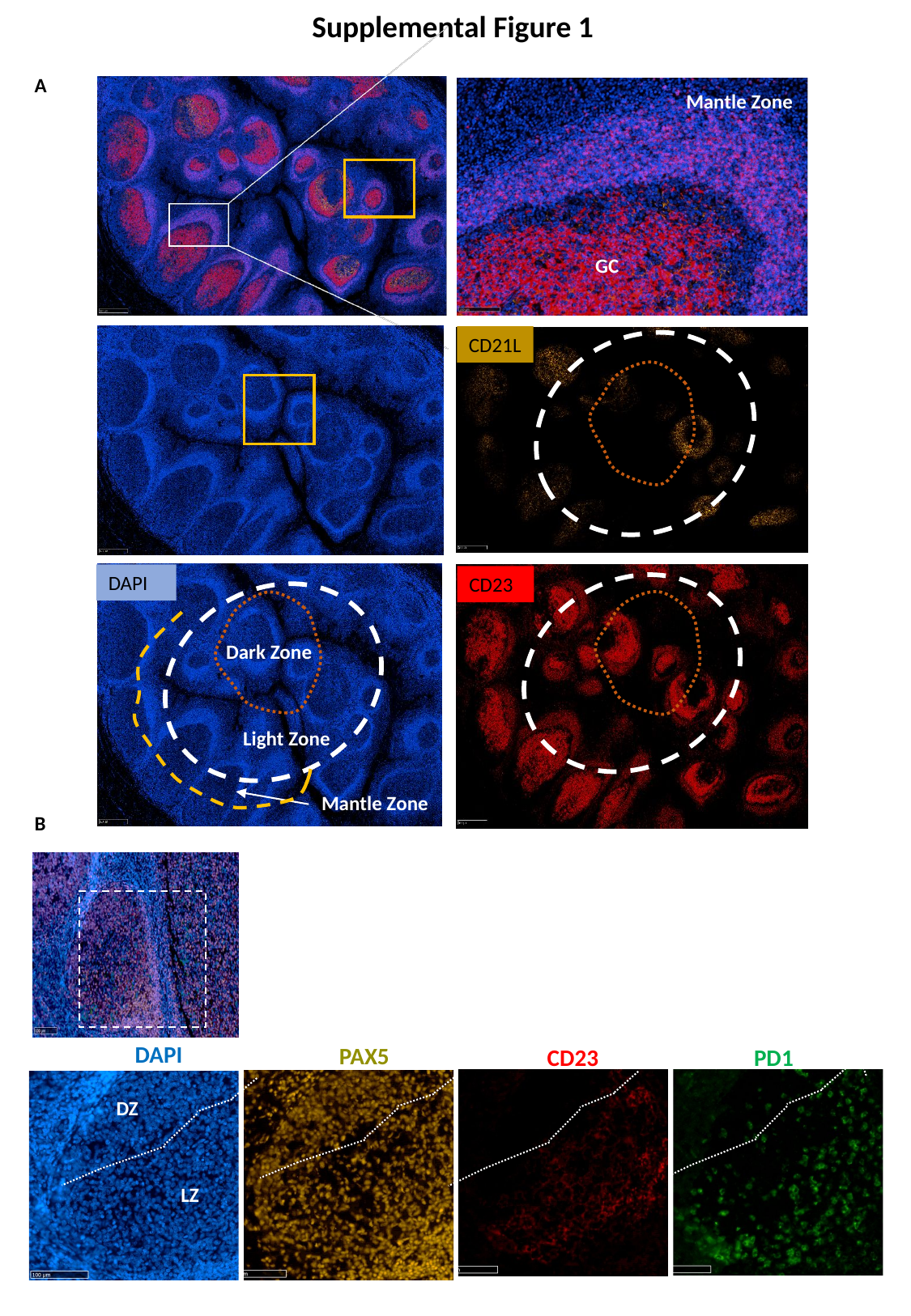

Supplemental Figure 1
Mantle Zone
GC
CD21L
Dark Zone
Light Zone
Mantle Zone
DAPI
CD23
Light Zone
A
B
DAPI
PAX5
CD23
PD1
DZ
LZ

## Slide 2
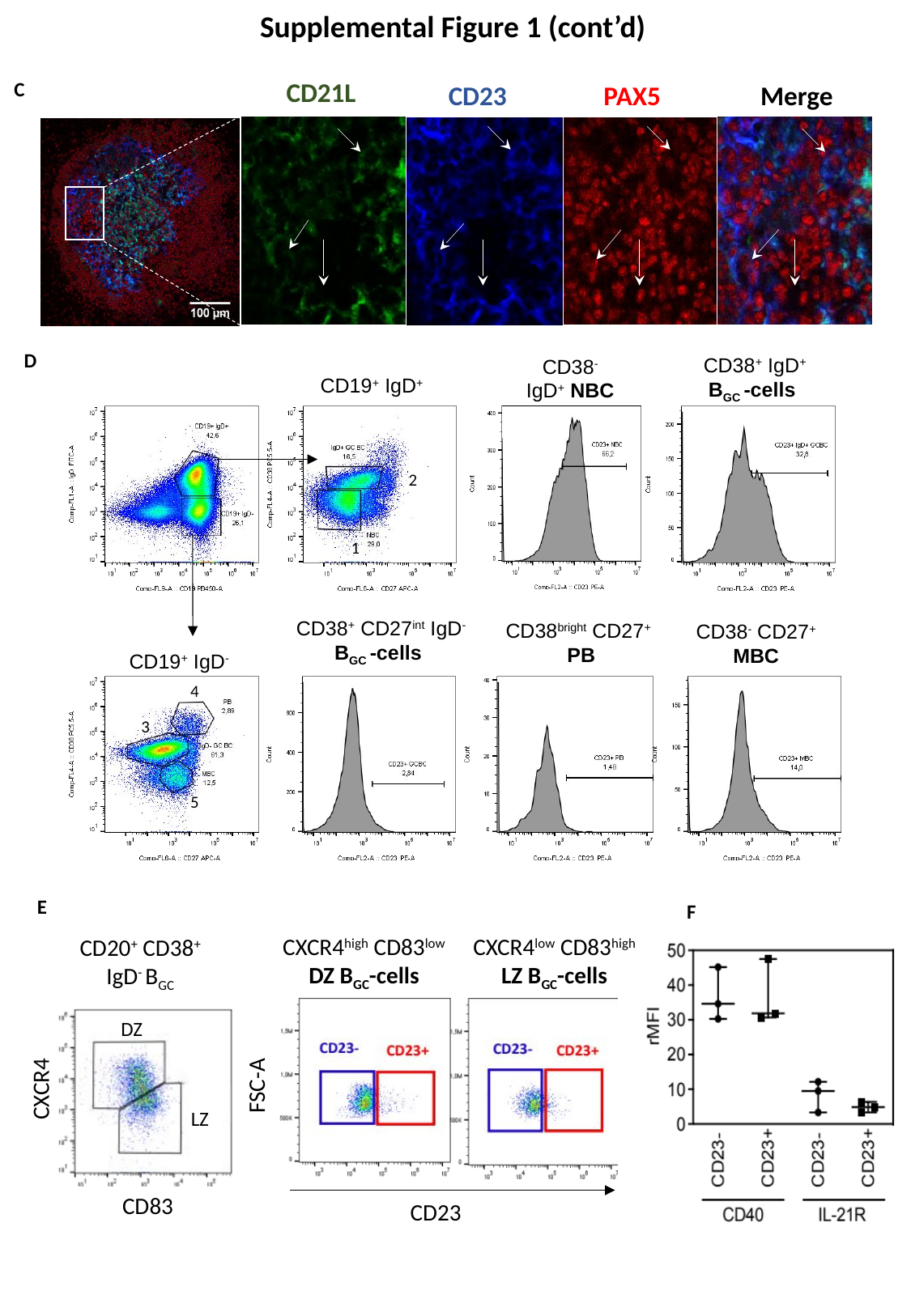

Supplemental Figure 1 (cont’d)
CD21L
C
PAX5
Merge
CD23
D
CD38+ IgD+
BGC -cells
CD38- IgD+ NBC
CD19+ IgD+
2
1
CD38+ CD27int IgD-
BGC -cells
CD38bright CD27+
PB
CD38- CD27+
MBC
CD19+ IgD-
4
3
5
E
F
CXCR4high CD83low
DZ BGC-cells
CXCR4low CD83high
LZ BGC-cells
CD20+ CD38+
 IgD- BGC
DZ
CXCR4
FSC-A
LZ
CD83
CD23

## Slide 3
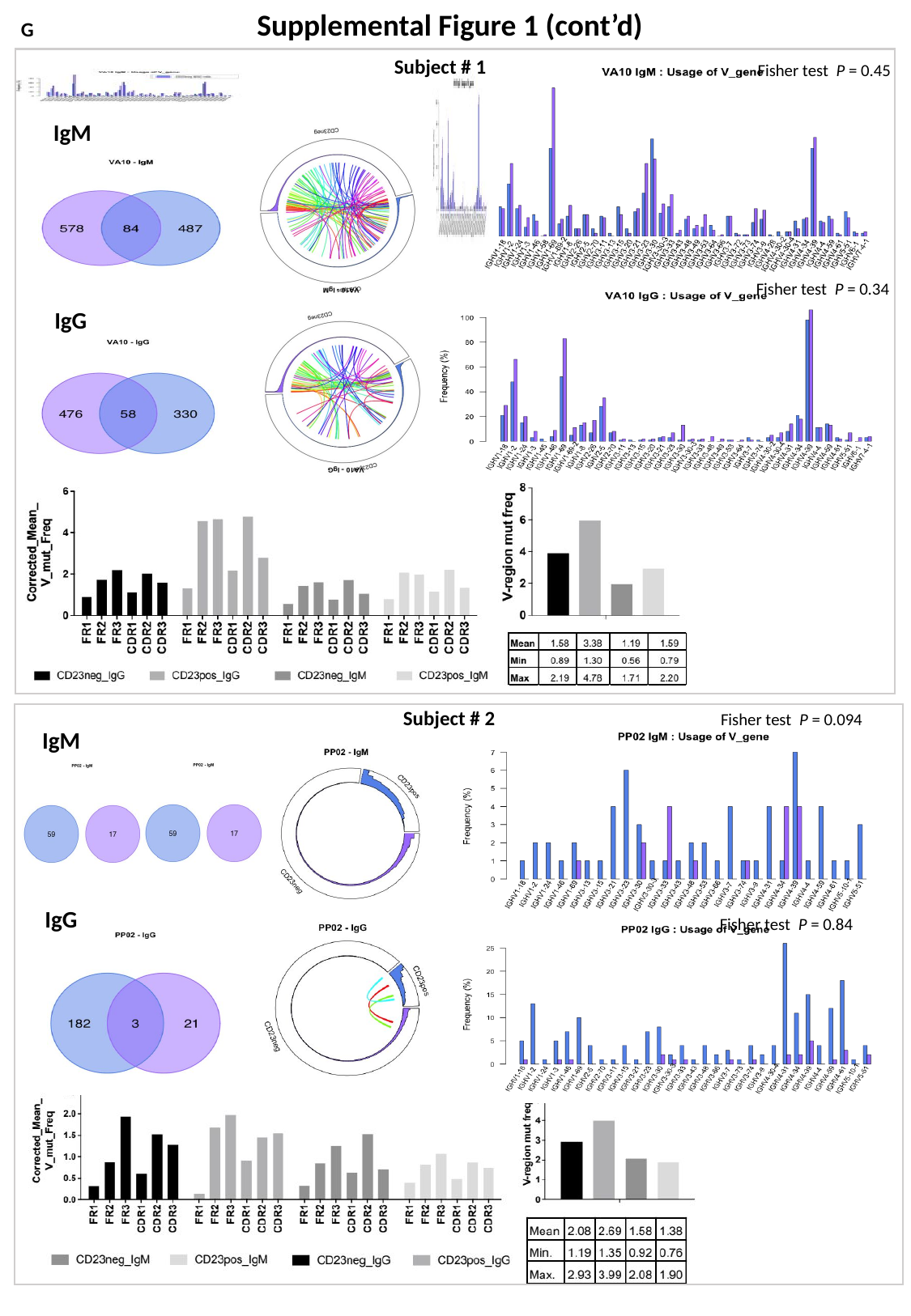

Supplemental Figure 1 (cont’d)
G
Subject # 1
Fisher test P = 0.45
IgM
Fisher test P = 0.34
IgG
Subject # 2
Fisher test P = 0.094
IgM
IgG
Fisher test P = 0.84

## Slide 4
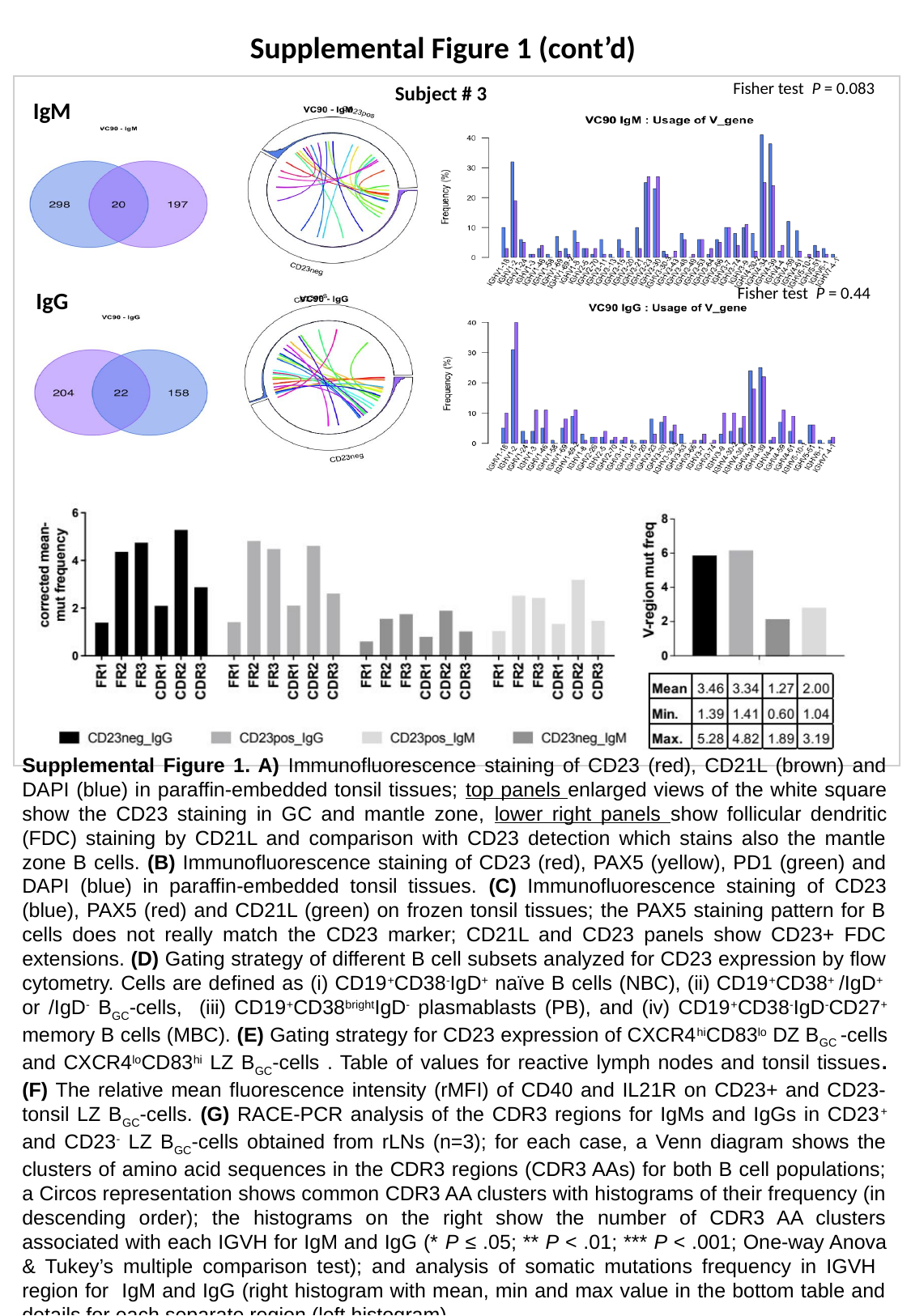

Supplemental Figure 1 (cont’d)
Fisher test P = 0.083
Subject # 3
IgM
Fisher test P = 0.44
IgG
Supplemental Figure 1. A) Immunofluorescence staining of CD23 (red), CD21L (brown) and DAPI (blue) in paraffin-embedded tonsil tissues; top panels enlarged views of the white square show the CD23 staining in GC and mantle zone, lower right panels show follicular dendritic (FDC) staining by CD21L and comparison with CD23 detection which stains also the mantle zone B cells. (B) Immunofluorescence staining of CD23 (red), PAX5 (yellow), PD1 (green) and DAPI (blue) in paraffin-embedded tonsil tissues. (C) Immunofluorescence staining of CD23 (blue), PAX5 (red) and CD21L (green) on frozen tonsil tissues; the PAX5 staining pattern for B cells does not really match the CD23 marker; CD21L and CD23 panels show CD23+ FDC extensions. (D) Gating strategy of different B cell subsets analyzed for CD23 expression by flow cytometry. Cells are defined as (i) CD19+CD38-IgD+ naïve B cells (NBC), (ii) CD19+CD38+ /IgD+ or /IgD- BGC-cells, (iii) CD19+CD38brightIgD- plasmablasts (PB), and (iv) CD19+CD38-IgD-CD27+ memory B cells (MBC). (E) Gating strategy for CD23 expression of CXCR4hiCD83lo DZ BGC -cells and CXCR4loCD83hi LZ BGC-cells . Table of values for reactive lymph nodes and tonsil tissues. (F) The relative mean fluorescence intensity (rMFI) of CD40 and IL21R on CD23+ and CD23- tonsil LZ BGC-cells. (G) RACE-PCR analysis of the CDR3 regions for IgMs and IgGs in CD23+ and CD23- LZ BGC-cells obtained from rLNs (n=3); for each case, a Venn diagram shows the clusters of amino acid sequences in the CDR3 regions (CDR3 AAs) for both B cell populations; a Circos representation shows common CDR3 AA clusters with histograms of their frequency (in descending order); the histograms on the right show the number of CDR3 AA clusters associated with each IGVH for IgM and IgG (* P ≤ .05; ** P < .01; *** P < .001; One-way Anova & Tukey’s multiple comparison test); and analysis of somatic mutations frequency in IGVH region for IgM and IgG (right histogram with mean, min and max value in the bottom table and details for each separate region (left histogram).

## Slide 5
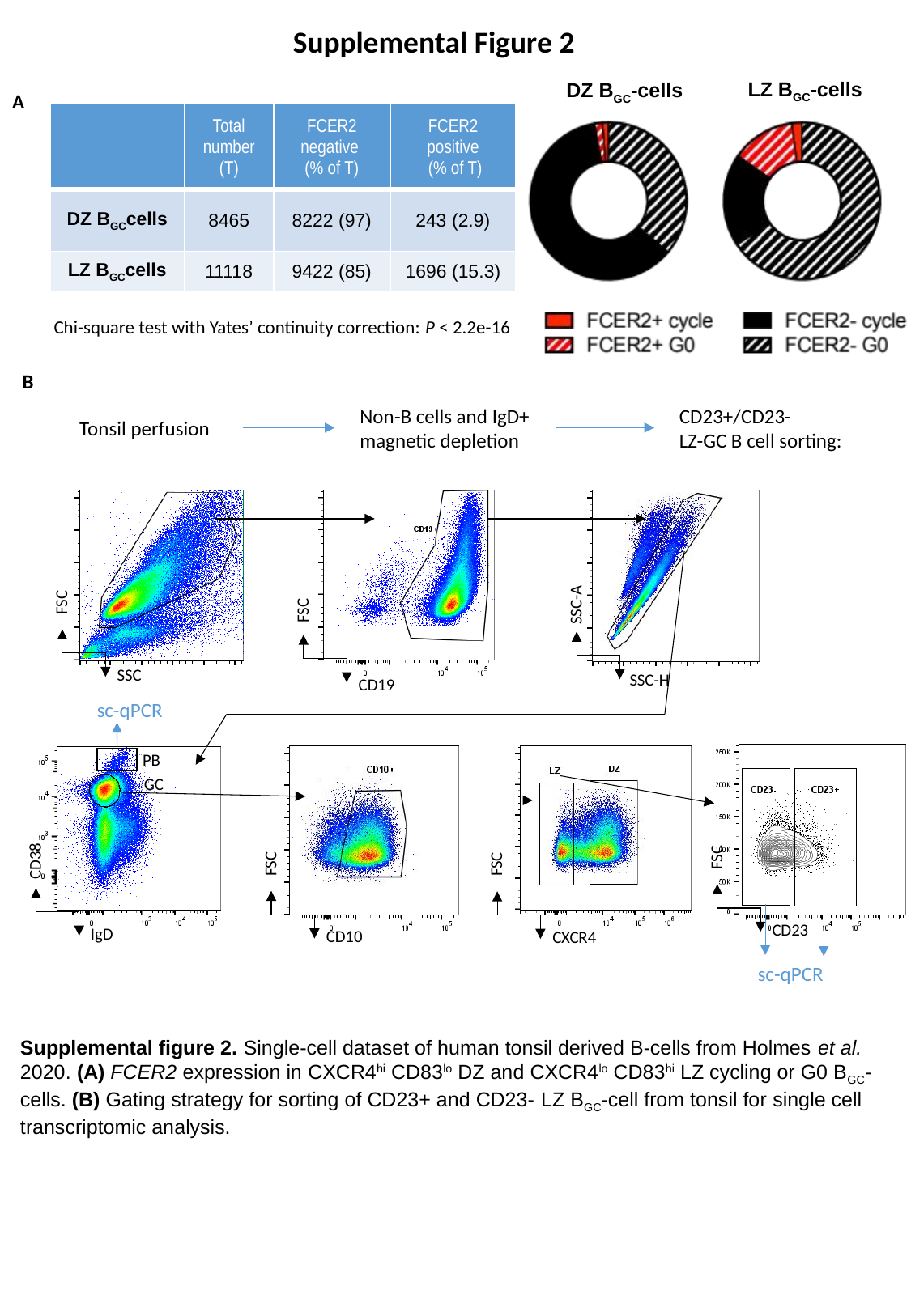

Supplemental Figure 2
LZ BGC-cells
DZ BGC-cells
A
| | Total number (T) | FCER2 negative (% of T) | FCER2 positive (% of T) |
| --- | --- | --- | --- |
| DZ BGCcells | 8465 | 8222 (97) | 243 (2.9) |
| LZ BGCcells | 11118 | 9422 (85) | 1696 (15.3) |
Chi-square test with Yates’ continuity correction: P < 2.2e-16
B
Non-B cells and IgD+
magnetic depletion
CD23+/CD23-
LZ-GC B cell sorting:
Tonsil perfusion
FSC
SSC-A
FSC
SSC
SSC-H
CD19
sc-qPCR
PB
GC
FSC
CD38
FSC
FSC
CD23
IgD
CD10
CXCR4
sc-qPCR
Supplemental figure 2. Single-cell dataset of human tonsil derived B-cells from Holmes et al. 2020. (A) FCER2 expression in CXCR4hi CD83lo DZ and CXCR4lo CD83hi LZ cycling or G0 BGC-cells. (B) Gating strategy for sorting of CD23+ and CD23- LZ BGC-cell from tonsil for single cell transcriptomic analysis.

## Slide 6
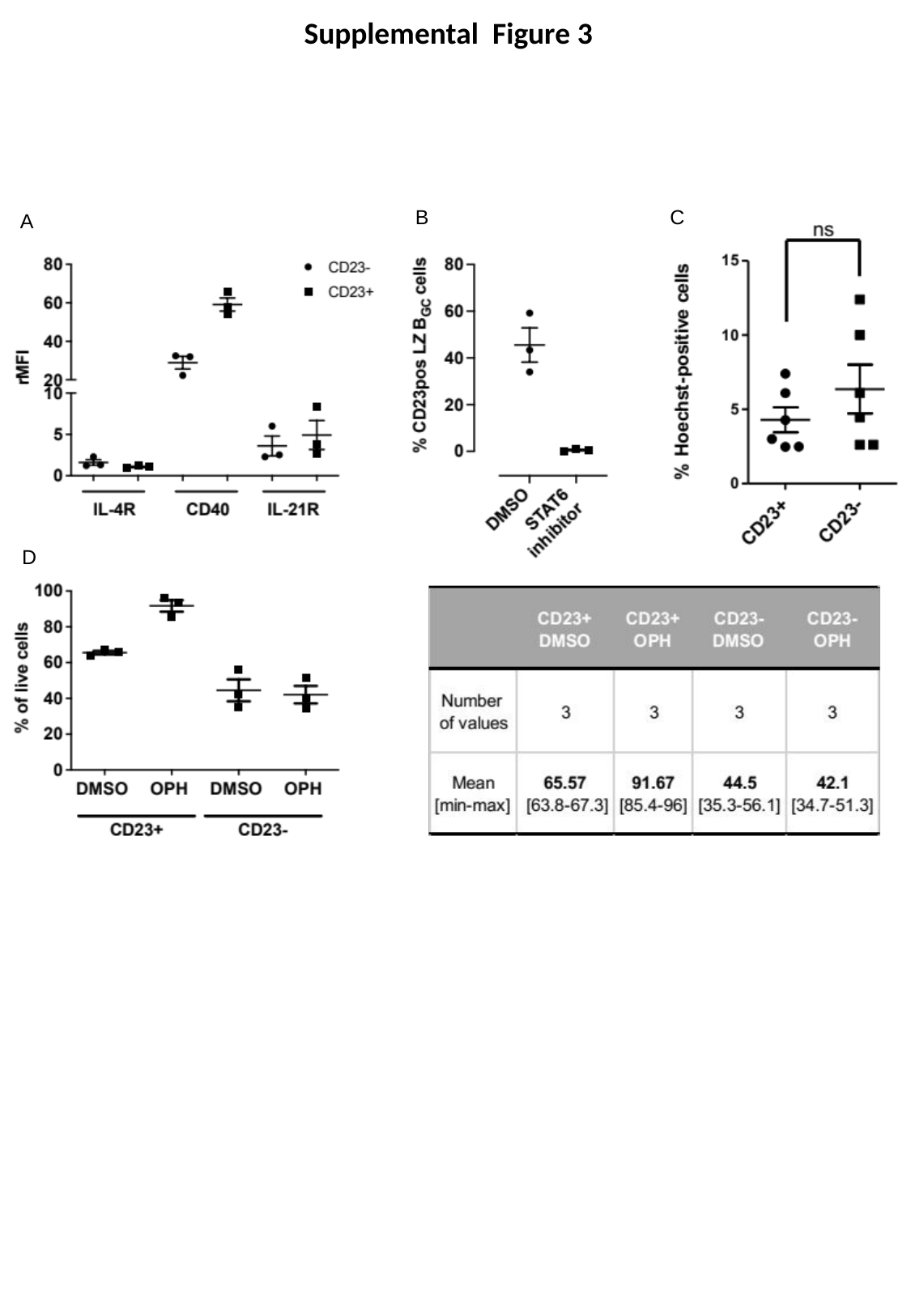

Supplemental Figure 3
B
C
A
D

## Slide 7
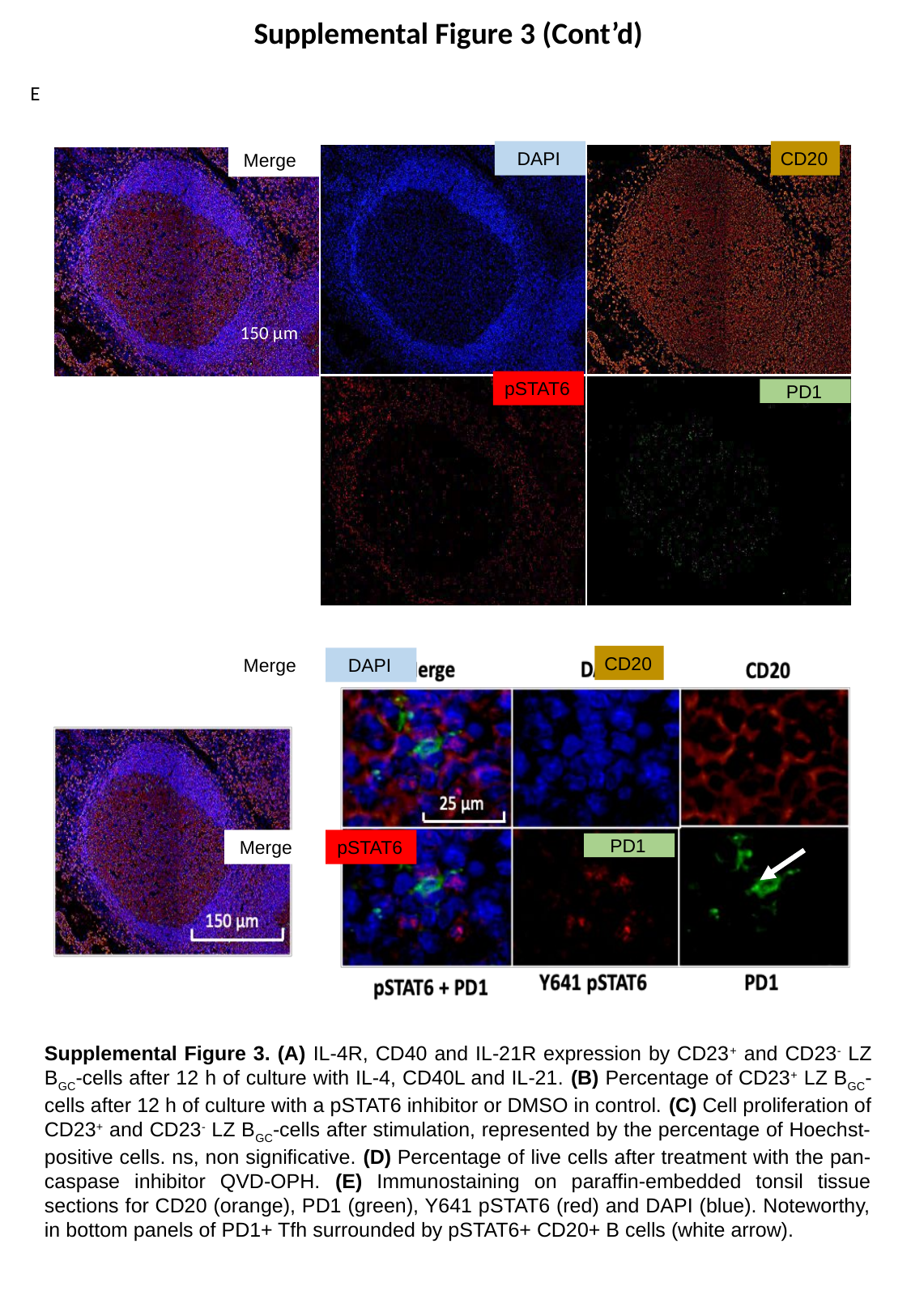

Supplemental Figure 3 (Cont’d)
E
DAPI
CD20
Merge
150 µm
pSTAT6
PD1
CD20
Merge
DAPI
PD1
Merge
pSTAT6
Supplemental Figure 3. (A) IL-4R, CD40 and IL-21R expression by CD23+ and CD23- LZ BGC-cells after 12 h of culture with IL-4, CD40L and IL-21. (B) Percentage of CD23+ LZ BGC-cells after 12 h of culture with a pSTAT6 inhibitor or DMSO in control. (C) Cell proliferation of CD23+ and CD23- LZ BGC-cells after stimulation, represented by the percentage of Hoechst-positive cells. ns, non significative. (D) Percentage of live cells after treatment with the pan-caspase inhibitor QVD-OPH. (E) Immunostaining on paraffin-embedded tonsil tissue sections for CD20 (orange), PD1 (green), Y641 pSTAT6 (red) and DAPI (blue). Noteworthy, in bottom panels of PD1+ Tfh surrounded by pSTAT6+ CD20+ B cells (white arrow).

## Slide 8
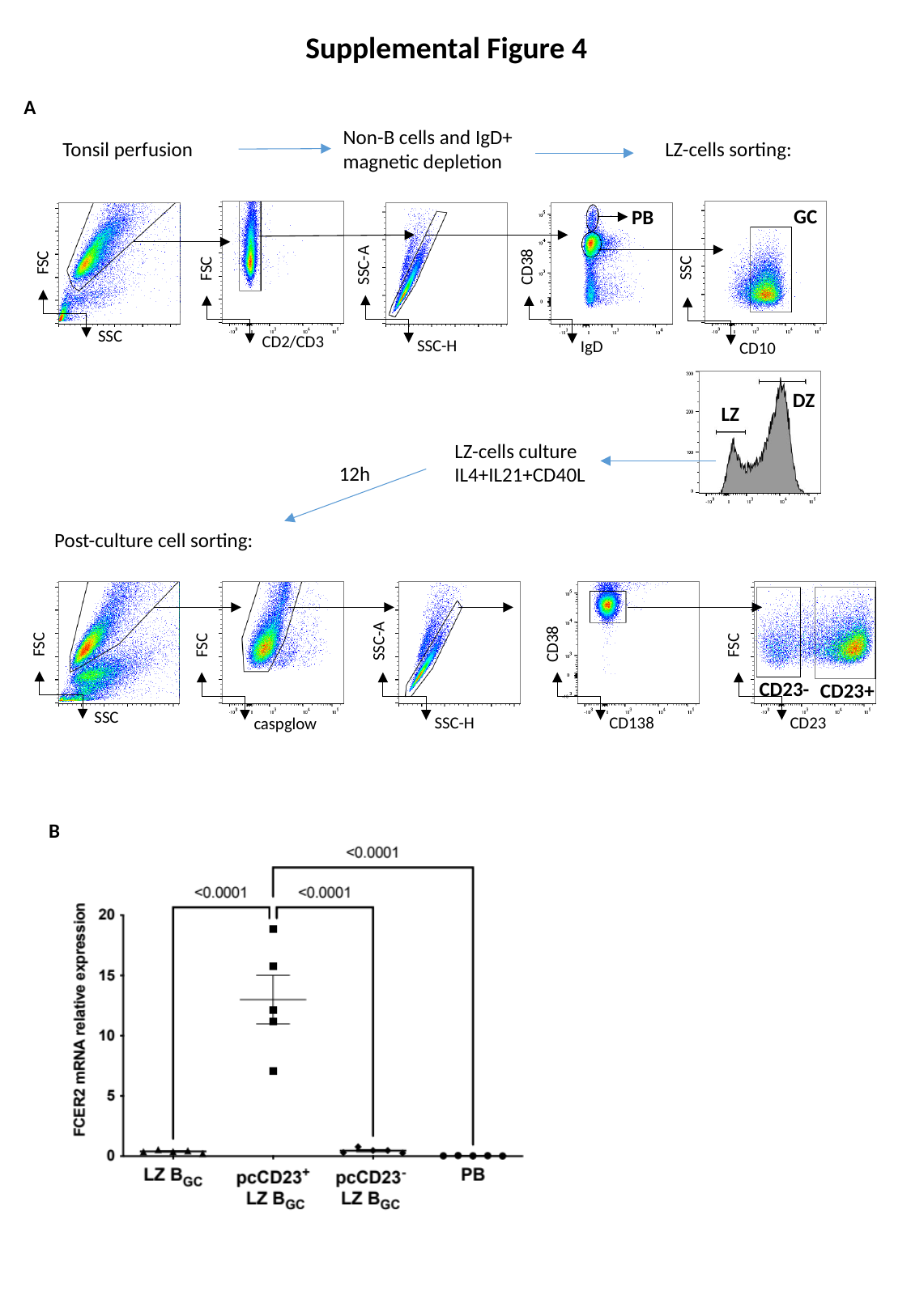

Supplemental Figure 4
A
Non-B cells and IgD+
magnetic depletion
LZ-cells sorting:
Tonsil perfusion
GC
PB
FSC
SSC
FSC
CD2/CD3
SSC-A
CD38
SSC
SSC-H
IgD
CD10
DZ
LZ
LZ-cells culture
IL4+IL21+CD40L
12h
Post-culture cell sorting:
FSC
SSC
SSC-A
CD38
FSC
FSC
CD23-
CD23+
CD138
CD23
SSC-H
caspglow
B

## Slide 9
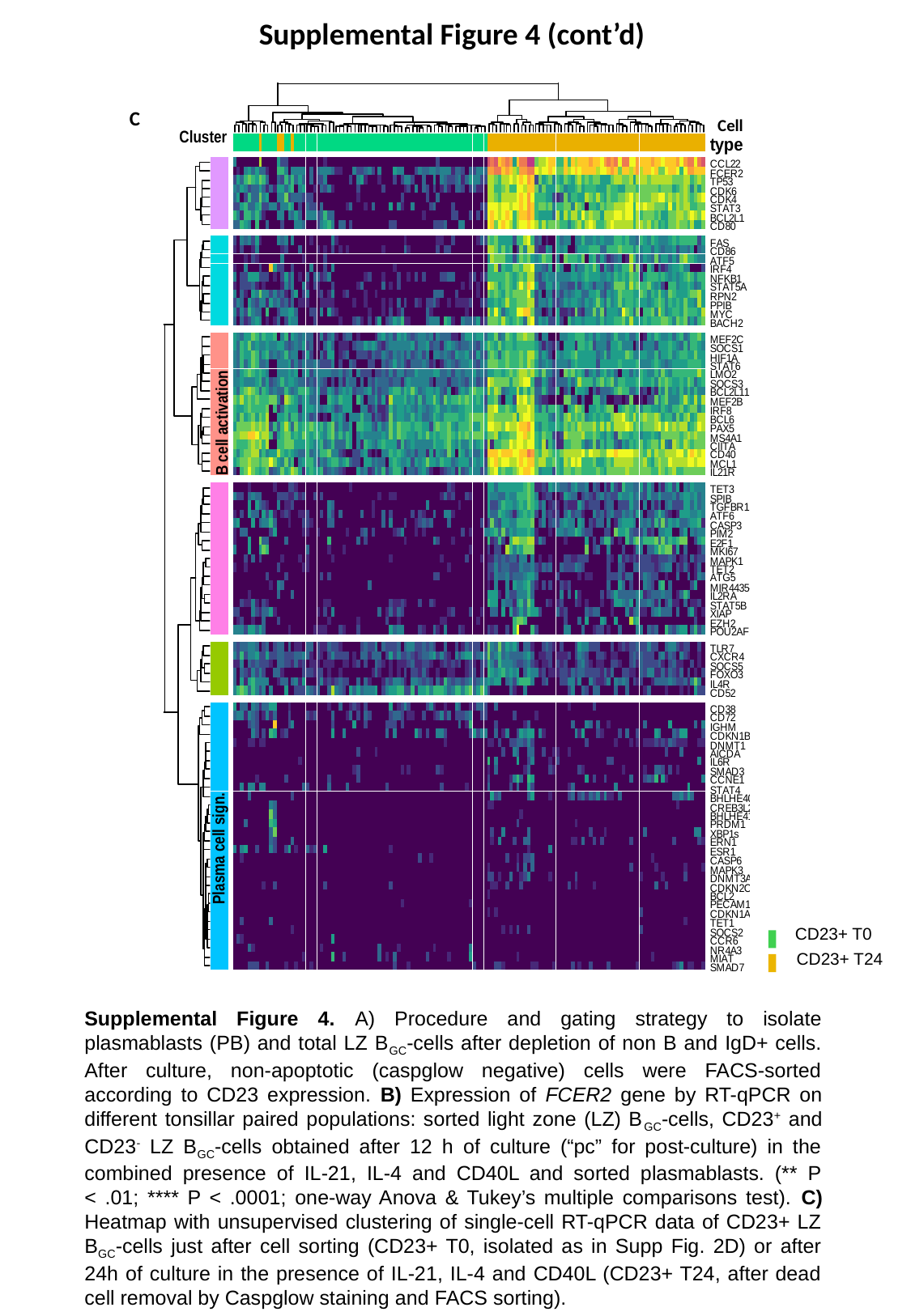

Supplemental Figure 4 (cont’d)
C
Cell
Cluster
B cell activation
Plasma cell sign.
CD23+ T0
CD23+ T24
Supplemental Figure 4. A) Procedure and gating strategy to isolate plasmablasts (PB) and total LZ BGC-cells after depletion of non B and IgD+ cells. After culture, non-apoptotic (caspglow negative) cells were FACS-sorted according to CD23 expression. B) Expression of FCER2 gene by RT-qPCR on different tonsillar paired populations: sorted light zone (LZ) BGC-cells, CD23+ and CD23- LZ BGC-cells obtained after 12 h of culture (“pc” for post-culture) in the combined presence of IL-21, IL-4 and CD40L and sorted plasmablasts. (** P < .01; **** P < .0001; one-way Anova & Tukey’s multiple comparisons test). C) Heatmap with unsupervised clustering of single-cell RT-qPCR data of CD23+ LZ BGC-cells just after cell sorting (CD23+ T0, isolated as in Supp Fig. 2D) or after 24h of culture in the presence of IL-21, IL-4 and CD40L (CD23+ T24, after dead cell removal by Caspglow staining and FACS sorting).

## Slide 10
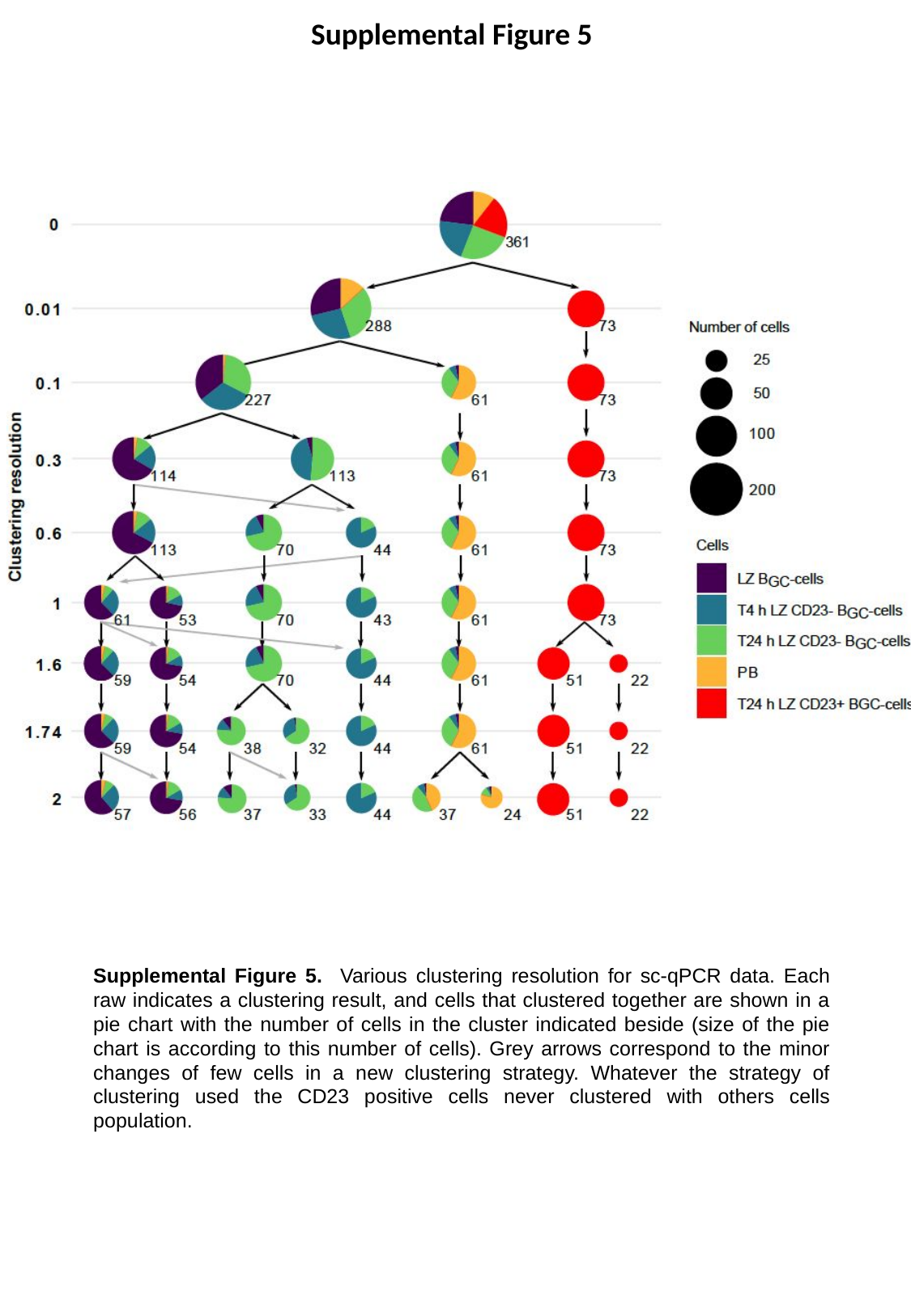

Supplemental Figure 5
Supplemental Figure 5. Various clustering resolution for sc-qPCR data. Each raw indicates a clustering result, and cells that clustered together are shown in a pie chart with the number of cells in the cluster indicated beside (size of the pie chart is according to this number of cells). Grey arrows correspond to the minor changes of few cells in a new clustering strategy. Whatever the strategy of clustering used the CD23 positive cells never clustered with others cells population.
