# Supplementary material for: Committed Human CD23-Negative Light-Zone Germinal Center B Cells Delineate Transcriptional Program Supporting Plasma Cell Differentiation
Source: Front Immunol. 2021 Dec 2;12:744573. doi: 10.3389/fimmu.2021.744573 (PMC8674954; doi:10.3389/fimmu.2021.744573)
Supplement: Supplementary file 2 [file DataSheet_2.docx]

**Supplemental Materials and Methods including Tables for Antibodies and Primers**

**Cell culture**

All cultures were performed in complete medium consisting of RPMI 1640 supplemented with 10% FCS and antibiotics ([All](https://www.thermofisher.com/us/en/home/brands/invitrogen.html) from Gibco, Life Technologies, Grand Island, NY). Tonsil-derived LZ B_GC_-cells were cultured at 0.8 x 10^5^ cells/ml and stimulated for 12 h or 24 h with 10 ng/ml recombinant human (rh) IL-4 ([R&D Systems](https://www.rndsystems.com/), Minneapolis, MN), 200 ng/ml soluble rhCD40L (Immunex) and 50 ng/mL rhIL-21 (Miltenyi Biotech). For RNA expression analysis, CD23- and CD23+ subsets were then cell-sorted after staining with Caspglow-FITC (to exclude apoptotic and dead cells), CD38 and CD138 (to exclude a potential contamination with PB or PC).

For differentiation experiments, CD23^+^ and CD23^-^ LZ B_GC_-cells obtained after 24h of culture with IL-4, CD40L and IL-21 were sorted by the BD FACS Aria and then cultured for 48 h with 50 U/ml rhIL-2 (Proleukin Novartis), 10 ng/ml rhIL-4 ([R&D Systems](https://www.rndsystems.com/)) and 12 ng/ml rhIL-10 (R&D Systems).

For cocultures between LZ B_GC_ cells and Tfh cells, autologous CD4^+^ T cells were purified by negative selection using magnetic cell separation with CD19, CD8, CD16 and CD14 purified mouse monoclonal antibodies (Beckman Coulter, Brea, CA) followed by anti-mouse microbeads (Miltenyi Biotech) with the AutoMACS deplete-sensitive program. Tfh cells (CD4^+^ CXCR5^+^ ICOS^+^ PD1^+^) were then sorted by the BD FACS Aria cell sorter. Tfh cells were added to LZ B_GC_-cells (ratio 1T:2B) with 0.2 μg/ml anti-CD3 plus 0.2 μg/ml anti-CD28 (PeliCluster Sanquin, Amsterdam, The Netherlands) or 250 µg/mL S*taphylococcal Enterotoxin B* (SEB, Sigma-Aldrich) during 24h.

For pSTAT6 inhibition, LZ B_GC_-cells were cultured with 1µM of pSTAT6 inhibitor (#AS1517499, Axon Medchem, Groningen, The Netherlands) or DMSO during 24 h in complete medium.

**Surface and intracellular staining for flow cytometry**

NBCs were defined as CD19^+^ IgD^+^ CD38^-^, MBCs as CD19^+^ CD38^-^ CD27^+^ and B_GC_-cells as CD19^+^ IgD^-^ CD38^+^.

For intracellular staining of pSTAT6, cells were fixed with 1.6% paraformaldehyde in complete medium during 10 minutes at room temperature. After a wash in PBS, cells were permeabilized with cold methanol during 15 min on ice. Cells were then labelled with STAT6(pY641)-Alexa 488 and CD23-PE antibodies for 15 min at 4°C. Data were acquired on a CytoFLEX flow cytometer (Beckman Coulter) and analyzed using FlowJo software.

For intracellular staining of KI67, cells were fixed and permeabilized using the cytofix/cytoperm buffer set (BD Biosciences). For intracellular staining of cMYC, p-S6, BCL6 and BLIMP1, cells were fixed and permeabilized using the Transcription Factor buffer set (BD Biosciences).

All monoclonal antibodies (mAbs) used are detailed in Supplemental Table I.

**qRT-PCR analysis**

RNA was extracted using NucloSpin RNA Plus XS Kit (Macherey-Nagel) and reverse transcribed into cDNA with Superscript II (Invitrogen). Multiplex qPCRs were performed using the TaqMan Gene Expression Master Mix (Applied Biosystems) and run on a 96.96 Dynamic Array IFC using the Fluidigm BioMark HD system. All TaqMan primers (Applied Biosystems) used in this study are listed in Supplemental table II. Gene expression levels were quantified using *ABL1* gene as endogenous control. The 2 exp(-∆∆Ct) method was used to determine the relative expression of each gene.

# For single-cell experiments expression data was imported in R version 3.6.2 with the Fluidigm SC package (version 3.6.2) (Sun and Wang, 2017). Quality control, data scaling, dimensionality reduction and clustering analysis were performed using the Seurat package (version 3.1.3) (Stuart *et al.,* 2019). Particularly, UMAP computation was executed on PCA coordinates using the following parameters: dims=1:5, n.neighbors = 15, min.dist= 0.45. Based on this UMAP results, Monocle3 package (version 0.2.0) was used to produce cell trajectory, which was ordered according to LZ B_GC_-cells and plasmablasts coordinates. Continuous gene expressions according to pseudotime were estimated by fitting generalized additive models with the mgcv R package (version 1.8.37) (Wood, 2010, https://doi.org/10.1111/j.1467-9868.2010.00749.x) and using the REML smoothing parameter estimation method.

# Graphical representations of clustering results and gene expression according to pseudotime were performed using ggplot2 (version 3.3.0) (Wickham *et al.,* 2016), pheatmap (version 1.0.12) (Kolde *et al.,* 2019) and clustree (version 0.4.3) (Zapia and Oshlack 2018, 10.1093/gigascience/giy083) packages.

**5 ́Rapid Amplification of cDNA Ends PCR (RACE PCR)**

A mix containing 500 ng mRNAs of CD23^+^ and CD23^-^ lymph node-derived LZ B_GC_-cells, 1µL of 10µM reverse primer complementary to IGHM, IGHG, IGL and IGK constant regions (5’-CGGGTRCTGCTGATGTCAGA-3’, 5’-GTGTTGCTGGGCTTGTGAT-3’, 5’-GACTTCGCAGGCGTAGACTT-3’, 5’-CTGGCCGCYTACTTGTTGTT-3’, respectively), and 1µL-deoxynucleoside 5’-triphosphate solution mix (New EnglandBiolabs), adjusted to a final volume of 12µL with water, was incubated for 3 min at 72°C and 2 mn at 42°C. After a short spin, the mix was placed on ice for 2 mn. Then, a mix of 4µL ProtoScript buffer, 2µL dithiothreitol solution, 1µL ProtoScriptII (New England Biolabs), and 1µL cap-race primer (5’-AAGCAGTGGTATCAACGCAGAGTACAT[GGGG]-3’, with the 4 G in brackets are ribonucleotides) was incubated 90 min at 42°C and 10 min at 70°C. At the end of the reaction, 20µL of RNAse free water was added.

The cDNA was amplified with TaqPhusion (New England Biolabs), using a universal forward primer mix (5’-CTAATACGACTCACTATAGGGC-3’ and 5’-CTAATACGACTCACTATAGGGCAAGCAGTGGTATCAACGCAGAGT-3’, in a ratio of 4:1) and a custom reverse primer specific of IGHM IGHG, IGL and IGK constant exons (5’-GTCTCGTGGGCTCGGAGATGTGTATAAGAGACAGCTCGTATCCGACGGGGAATT-3’, 5’-GTCTCGTGGGCTCGGAGATGTGTATAAGAGACAGCGGTTCGGGGAAGTAGTCC-3’, 5’-GTCTCGTGGGCTCGGAGATGTGTATAAGAGACAGGCGGGAAGATGAAGACAGAT-3’, 5’-GTCTCGTGGGCTCGGAGATGTGTATAAGAGACAGGTTGGCTTGYAGCTCCTCAG-3’). Each PCR was performed in four replicates. Cycling conditions were 30 s at 98°C, 32 cycles of 30 s at 98°C, 30 s at 65°C, 30 s at 72°C, with a final elongation of 5 min at 72°C. The 4 replicates were pooled and migrated on an agarose gel, and the fragments of interest were purified using the kit Nucleospin gel and PCR clean-up kits (Macherey-Nagel). Then, Illumina sequencing adapter and tag sequences were added by primer extension using Taq Phusion (New England Biolabs). Samples were divide into 4 replicates and PCR was performed using unique tags for each replicates. Cycling conditions were 30 s at 98°C, 12 cycles of 30 s at 98°C, 30 s at 65°C, 30 s at 72°C, and final elongation 5 mn at 72°C. The 4 replicates were mixed and gel purified as previously.

Resulting amplicons were sequenced on an Illumina MiSeq sequencing system using MiSeq Reagent Kit V3 600 cycles Illumina). Paired reads were merged using FLASH software. Repertoire analysis was performed using IMGT/HighV-QUEST tool (http://imgt.org/) for sequence alignments, annotations and mutations frequency and amino acid (AA) changes are depicted for each region. We developed an inhouse R script to identify clonotype. Briefly, reads were considered to originate from the same clonotype if they share the same V and J genes and a significant similarity in their CDR3 sequence. To define the extent of variability allowed between CDR3, each CDR3 AA sequence was aligned against itself to define a maximum alignment score. Then all CDR3 from reads bearing the same V and J genes were aligned against each other. CDR3 were considered as coming from the same clonotype if their alignment score was superior or equal to 70% of their maximum alignment score, and grouped together.

The B cell repertoire was studied for each IgM and IgG libraries for each patient. The Venn diagram shows distribution of unique and overlapping CD23+ and CD23- CDR3 AA clonotypes (VennDiagram R package v1.6.20). The CDR3 AA clonotypes in common between IgG and IgM for each patient are represented in a Circos plot (circlize R package v0.4.10). The histogram track, displayed for each population, illustrates the relative proportion of the number of sequences representing each CDR3 AA clonotype as a fraction of the total number of sequences for a sample. V-gene usages complement the characterization of each repertoire. The Fisher exact test was used to compare V-gene usage between the two populations for each repertoire.

**Supplemental Table 1**: Flow cytometry antibodies list

**Supplemental Table 2 :** List of TaqMan Assay-on-Demand^TM^**.**

**Supplemental Table 3**: list of antibodies for immunohistofluorescence

| **Antibody target**  **(species raised in)** | **Fluorochrome** | **Manufacturer** | **Reference** | **Dilution** | |
| --- | --- | --- | --- | --- | --- |
| Anti-PAX5 | Unconjugated | Abcam | Ab109443 | 1/100 |  |
| Anti-CD3 | Unconjugated | Abcam | Ab11089 | 1/100 |  |
| Anti-CD20 | Unconjugated | Abcam | Ab78237 | 1/100 |  |
| Anti-CD23 | Unconjugated | Abcam | EPR3617 | 1/100 |  |
| Anti-CD21L | Unconjugated | Dako Cytomation |  | 1/100 |  |
| Anti-CXCL12 | Unconjugated | Thermofisher | 14-7992-81 | 1/100 |  |
| Anti-Mouse | Alexa-Fluor 647 | Jackson ImmmunoResearch | 715-606-151 | 1/500 |  |
| Anti IgM Mouse | Alexa-Fluor 594 | Jackson ImmmunoResearch | 715-606-020 | 1/500 |  |
| Anti-Rabbit | Alexa-Fluor 488 | Jackson ImmmunoResearch | 711-546-152 | 1/500 | |
| Anti-Rabbit | Alexa-Fluor 647 | Jackson ImmmunoResearch | 711-606-152 | 1/500 | |
| Anti-Rabbit | Alexa-Fluor 594 | Jackson ImmmunoResearch | 711-586-152 | 1/500 | |
| Anti-Rat | Alexa-Fluor 488 | Jackson ImmmunoResearch | 712-546-153 | 1/500 | |

**Supplemental Table 4**: list of antibodies for multiplex immunofluorescence detection on paraffin-embedded tissues

**Supplemental Table 5:** Gene prediction and biological network integration of our list of genes from scqPCR identified clusters with predicted related genes using GeneMANIA prediction server (Warde-Farley D et al., Nucleic Acids Res. 2010 )

**Supplemental table 6:** List of genes significantly differentially expressed between CD23 positive and CD23 negative cells.

| **Gene** | **avg_logFC  CD23neg-vs-CD23pos** | **p_val** |
| --- | --- | --- |
| **CD86** | -0.6959 | 0.0005 |
| **FCER2** | -2.0502 | 0.0008 |
| **STAT4** | -1.2530 | 0.0023 |
| **SOCS5** | -1.2375 | 0.0052 |
| **MAPK1** | -0.4617 | 0.0072 |
| **CCNE1** | 1.1354 | 0.0140 |
| **HIF1A** | -0.5606 | 0.0175 |
| **MKI67** | 0.9568 | 0.0200 |
| **CD38** | 0.3717 | 0.0281 |
| **CDK6** | 0.0570 | 0.0306 |
| **NFKB1** | -0.8769 | 0.0354 |
| **E2F1** | 0.4777 | 0.0365 |
| **TET2** | -0.6142 | 0.0385 |
| **ICOSL** | 0.4261 | 0.0447 |
| **DNMT1** | 0.6425 | 0.0464 |
